# A gonogenic stimulated transition of mouse embryonic stem cells with enhanced control of diverse differentiation pathways

**DOI:** 10.1038/srep25104

**Published:** 2016-05-09

**Authors:** Cameron Moshfegh, Lina Aires, Malgorzata Kisielow, Viola Vogel

**Affiliations:** 1 ETH Zurich, Department of Health Sciences and Technology, Laboratory of Applied Mechanobiology Vladimir Prelog-Weg 4, CH-8093 Zurich, Switzerland; 2 ETH Zurich, Flow Cytometry Core Facility, Otto-Stern-Weg 7, CH-8093 Zurich, Switzerland

## Abstract

Embryonic stem (ES) cells share markers with undifferentiated primordial germ cells (PGCs). Here, we discovered that a cellular state with some molecular markers of male gonocyte induction, including a G1/S phase arrest and upregulation of specific genes such as *Nanos2*, *Tdrd1*, *Ddx4*, *Zbtb16* and *Plk1s1*, can be chemically induced in male mouse ES cells *in vitro*, which we termed gonogenic stimulated transition (GoST). After longer culture of the resulting GoST cells without chemical stimulation, several molecular markers typical for early gonocytes were detected including the early gonocyte marker Tex101. Motivated by previous studies that found multipotency in cell lines derived from neonatal male germ cells *in vitro*, we then compared the differentiation potential of GoST cells to that of ES cells *in vitro*. Interestingly, GoST cells showed equal neurogenic, but enhanced cardiogenic and hepatogenic differentiation compared to ES cells *in vitro*. This work shows for the first time that some important molecular markers of the first developmental sexual differentiation program can be induced in male mouse ES cells *in vitro* and defines a novel concept to generate cells with enhanced multipotency.

Pluripotent stem (PS) cells hold considerable promise for applications in regenerative medicine. New methods to manipulate PS cell differentiation *in vitro* are of great interest^1^. Intriguingly, it was shown in the mouse that multipotent germ stem (mGS) cell lines could be derived from a subpopulation of neonatal male germ cells^2^,^3^. These mGS cells could differentiate into cells of all three germ layers similarly to PS cells *in vitro*, but showed partially pluripotent characteristics *in vivo*^2^,^3^. This raises the question whether the multilineage differentiation of mGS cells involves different mechanisms compared to that of PS cells.

Mouse embryonic stem (ES) cells represent an *in vitro* model for naive PS cells and can be used to study mammalian developmental processes^1^. Historically, the *in vitro* culture of mouse ES cells was established by adopting the culture conditions of embryonic carcinoma (EC) cells^4^,^5^, which were derived from germ cell tumors and found to be pluripotent^6^. Interestingly, naive ES cells, such as mouse ES cells *in vitro*, express several markers found in primordial germ cells (PGCs) from mouse embryonic days 8.5–12.5 (E8.5–12.5), such as *Dazl* and *Dppa3*^7^,^8^. It therefore seems possible that mouse ES cells *in vitro* may share more similarity with PGCs than is currently assumed^9^. PGCs represent the first cells of the germline and are induced in the epiblast by bone morphogenetic protein (BMP) signaling from the extraembryonic ectoderm at E6.25^10^ (Fig. 1A). They experience a genome wide demethylation (E8.5–13.5) and migrate towards the genital ridge (E8.5–11.5) where they undergo a sexually dimorphic differentiation into male and female gonocytes (E12.5–13.5), described as gonocyte induction^10^,^11^ (Fig. 1A). These gonocytes show a distinct pattern of molecular markers during their development (Fig. 1A). The nuclear protein TRA98 is expressed by preimplantation embryos and the germline including PGCs and gonocytes^7^,^12^,^13^. RNA-binding proteins, including Nanos2^14^,^15^,^16^, and cyclin-dependent kinase (CDK) inhibitors, including p27^17^, were shown to play important roles for the establishment of male gonocyte induction and are upregulated in differentiating PGCs from E12.5–13.5 onwards. The terminal carbohydrate epitope SSEA1 is expressed in PGCs and is downregulated from E12.5–15 onwards^18^,^19^,^20^,^21^,^22^, while the chemokine receptor Cxcr4 is expressed in migrating PGCs, but is downregulated from E13.5 onwards^23^. The membrane protein Tex101 is upregulated in gonocytes from E14–16 onwards^24^. Dppa3 is expressed in gonocytes until E15.5^7^,^25^ and gradually decreases in male gonocytes afterwards until it becomes no longer detectable at 1 day postpartum (1dpp)^7^. Other studies showed that essential regulators of germ cell competence and meiosis, such as the RNA-binding protein Dazl^8^,^11^, are also essential to maintain pluripotency in mouse ES cells^8^. Germ cell competence and pluripotency therefore seem to share common regulatory mechanisms, suggesting that the mechanisms regulating differentiation of PGCs could potentially be activated in ES cells.

In this work, we initially aimed to induce a proliferation arrest in mouse ES cells in the absence of leukemia inhibitory factor (LIF) and β-mercaptoethanol (βME) without loss of cell viability. Such a cellular state was achieved using a specific chemical treatment (Fig. 1B). Because the resulting cells expressed some markers specific for gonocyte induction (Fig. 1A), we defined this particular state as gonogenic stimulated transition (GoST) and the resulting cells as GoST cells (Fig. 1B). After making this unexpected discovery, our subsequent goal was to characterize GoST cells for more markers of gonocyte induction and for their multilineage differentiation potential in order to establish a first knowledge basis supporting future studies of their potential for stem cell therapy.

## Results

**Figure 1:**
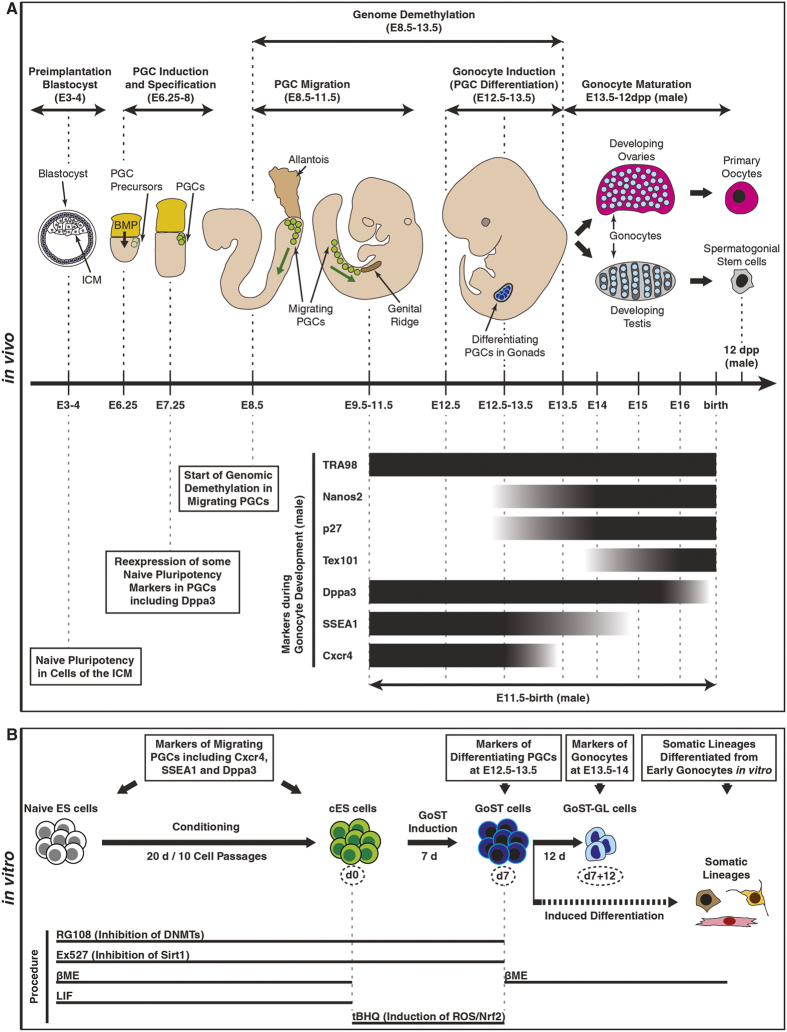
Gonocyte induction during embryogenesis *in vivo* and GoST induction *in vitro*. (**A**) A schematic representation of germ cell development during mouse embryogenesis *in vivo*. PGCs are induced in the epiblast by BMP signaling at E6.25 and their specification is completed by E7.25. PGCs migrate from E8.5–11.5 towards the genital ridge, while their genome is demethylated from E8.5–13.5. PGCs arrest and differentiate within the genital ridge into gonocytes, described here as gonocyte induction from E12.5–13.5. Gonocytes mature within the developing ovaries and testis from E13.5 onwards and ultimately differentiate into the primary oocytes and spermatogonial stem cells, respectively. Green arrows indicate the direction of PGC migration. Drawings were created from observation of mouse embryos (http://www.emouseatlas.org)^53^. Markers expressed by the germline during male gonocyte development are shown for the time period of E11.5-birth. TRA98 is expressed by the germline at all time points^7^,^12^,^13^. Expression of Nanos2^14^,^15^,^16^ and p27^17^ is upregulated in differentiating PGCs during gonocyte induction from E12.5–13.5 onwards. Tex101 is upregulated in gonocytes from E14–16 onwards^24^. Dppa3 is expressed in gonocytes until E15.5^7^,^25^ and gradually decreases in male gonocytes afterwards until it becomes no longer detectable at 1 day postpartum (1dpp)^7^. SSEA1 is expressed in PGCs and is downregulated from E12.5–15 onwards^18^,^19^,^20^,^21^,^22^. Cxcr4 is expressed in migrating PGCs, but is downregulated from E13.5 onwards^23^. (**B**) A schematic representation of GoST induction and the treatment procedure *in vitro*. cES (green) cells are obtained by culturing ES cells (white) in the presence of LIF, βME, RG108 (inhibition of DNMTs) and Ex527 (inhibition of Sirt1) while passaging the cells every 2 days for 10 cell passages (20 days). GoST cells (dark blue) are obtained by culturing cES cells without LIF and without βME, but in the presence of tBHQ (induction of ROS and Nrf2), RG108 and Ex527 for 7 days. Further culture of GoST cells resulted in cells with markers of early gonocytes (GoST-GL cells). Markers of the relevant developmental time points listed in Fig. 1A are indicated for the cells obtained during the *in vitro* process in Fig. 1B. GoST cells could also be induced towards multiple somatic lineages.

We aimed to induce a proliferation arrest in mouse ES cells without loss of cell viability in the absence of LIF and βME. While LIF supports the naive state of pluripotency, βME is essential for ES cell culture by acting as an external antioxidant. LIF and βME were removed to prevent interference with potential signaling different from that in ES cells. A test of different combinations of chemicals revealed that cell viability was best preserved by a two-step chemical treatment procedure (Fig. 1B and S1). In the first step, conditioned ES (cES) cells were generated by culturing ES cells in the presence of the DNA methyltransferase (DNMT) inhibitor RG108 to reduce genomic methylation and the Sirtuin1 (Sirt1) inhibitor Ex527 to inhibit Sirt1 deacetylase activity, together with LIF and βME (Fig. 1B). In the second step (GoST induction), GoST cells were generated by culturing cES cells with the electrophilic redox-cycling compound and nuclear factor erythroid 2-related factor 2 (Nrf2) activator tBHQ, in addition to RG108 and Ex527 but without LIF and βME (Fig. 1B) (see Supplementary information for rationale of GoST induction).

### Upon GoST induction cells survive in conditions deprived of LIF and βME

**Figure 2:**
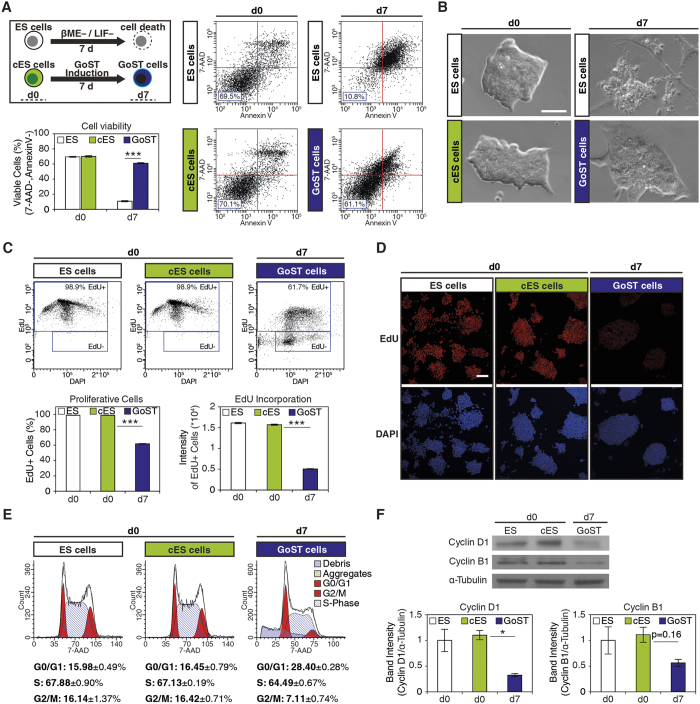
GoST cells survive in conditions deprived of LIF and βME and show decreased proliferation with a G1/S phase arrest compared to ES cells. (**A**) Flow cytometric analysis of cell viability by 7-AAD/Annexin V staining for cES cells (green), GoST cells (dark blue), ES cells (white) and ES cells subjected to 7 days of LIF and βME withdrawal (white). Viable cells are located in the lower left quadrant of 7-AAD/Annexin V plots. After 7 days, GoST cells showed considerably higher cell viability (61.1%) compared to ES cells (10.8%). (**B**) Phase contrast images of cell colonies. At day 7 of LIF and βME withdrawal ES cells were mostly dead and only very flat or elongated cells remained, while GoST cells at day 7 could still be observed in cellular colonies with similar morphology to ES cells at day 0. Scalebar = 50 μm. (**C**) Dual parameter plot of EdU incorporation (6 hour pulse of EdU) and DNA content (DAPI). The fraction of EdU + cells was lower upon GoST induction (61.7%) compared to ES cells and cES cells (98.9%). EdU + cells had a 3times lower mean EdU intensity upon GoST induction, compared to EdU + ES cells or cES cells (shown in the lower right bar chart). (**D**) Fluorescence images of EdU incorporation (red). DNA was counterstained with DAPI (blue). Upon GoST induction, the EdU signal intensity was decreased, while some cells within as well as outside the colonies showed no EdU signal. Scalebar = 100 μm. (**E**) Flow cytometric analysis of cell cycle distribution by measurement of DNA content. Upon GoST induction, the G0/G1 phase was increased and the G2/M phase was decreased which implies a G1/S-phase arrest. DNA was stained with 7AAD. (**F**) Western blot analysis of cell cycle regulatory proteins. Upon GoST induction, expression of Cyclin D1 and Cyclin B1 was decreased. Western blot data were normalized to α-Tubulin. Cell viability, EdU incorporation, Cell cycle and Western blot data were generated from three independent experiments. Error bars correspond to S.E.M. One star represents p < 0.05 and three stars represent p < 0.001. See Figs S1 and S2.

To investigate the nature of GoST cells, we compared cell viability of GoST cells and ES cells which were cultured in the absence of LIF and βME for 7 days by a dual staining with 7-AAD and Annexin V (Fig. 2A). While ES cells deprived of LIF and βME showed massive cell death (10.8% cell viability), cell viability of GoST cells was mostly maintained (61.1% cell viability) compared to that of cES cells (70.1% cell viability) and ES cells grown in the presence of LIF and βME (69.5% cell viability) (Fig. 2A). In ES cell cultures deprived of LIF and βME, most surviving cells had an elongated cell shape, indicating differentiation (Fig. 2B). In contrast, most cells in GoST cell cultures still showed a similar morphology to undifferentiated ES cells (Fig. 2B). It was shown in another study that the culture of ES cells is not possible without an external antioxidant^26^. Our finding that GoST cells survived in the absence of βME implied that they were not ES cells.

tBHQ is known to activate Nrf2 signaling, which mainly promotes cell survival^27^ (Fig. S2). Therefore, we analyzed the expression of Nrf2 target genes involved in antioxidant and NADPH generation systems (Fig. S2). As expected, expression of all Nrf2 target antioxidant genes and most target genes involved in NADPH generation was increased upon GoST induction (Fig. S2). Immunofluorescence of Nrf2 showed that after 2 days of GoST induction, Nrf2 was depleted in the cytoplasm of GoST cells compared to ES cells grown without LIF and βME for 2 days, indicating cytoplasmic-to-nuclear translocation of Nrf2 in GoST cells (Fig. S2). Altogether, these results implied that survival of GoST cells in βME-deprived conditions was rescued by activation of Nrf2 target genes (see Supplementary information for analysis of metabolic markers).

### Cell proliferation is reduced in GoST cells

We next analyzed cell proliferation by measuring incorporation of EdU (Fig. 2C,D) and additionally by quantification of cyclin D1 and B1 expression levels (Fig. 2F). ES cells and cES cells displayed very high and indistinguishable levels of EdU incorporation (98.9% EdU + cells) and appeared to be arrested in the G1 phase (Fig. 2C) due to toxicity from high EdU incorporation. This implied that ES cells and cES cells had very high rates of proliferation (Fig. 2C,D). In contrast, upon GoST induction, cell cycle progression in the presence of EdU was still observed, but the number of cells incorporating EdU was lower (61.7% EdU + cells) (Fig. 2C). Additionally, EdU + GoST cells showed a 3-times lower mean EdU intensity, compared to EdU + ES cells or cES cells (Fig. 2C). Altogether, these results implied that upon GoST induction, the proliferative cell fraction was reduced and that proliferating cells exhibited a reduced rate of DNA synthesis. Confirming this observation, expression of cyclin D1 and B1 was reduced upon GoST induction (Fig. 2F).

### GoST cells experience a G1/S phase arrest with upregulation of the G1/S phase-specific CDK inhibitor p27

Cell cycle distribution was analyzed by measuring the DNA content of cells with a 7-AAD staining (Fig. 2E). ES cells and cES cells showed the typical cell cycle distribution reported for ES cells^28^ with around 16% in G0/G1 phase, 16% in G2/M phase and 67% in S phase (Fig. 2E). Interestingly, GoST cells showed quite a distinct cell cycle distribution with around 28% in G0/G1 phase, 7% in G2/M phase and 64% in S phase (Fig. 2E). Upon GoST induction, the G0/G1 phase was increased while the G2/M phase was decreased, which implied the activation of the G1/S cell cycle checkpoint (Fig. 2E).

**Figure 3:**
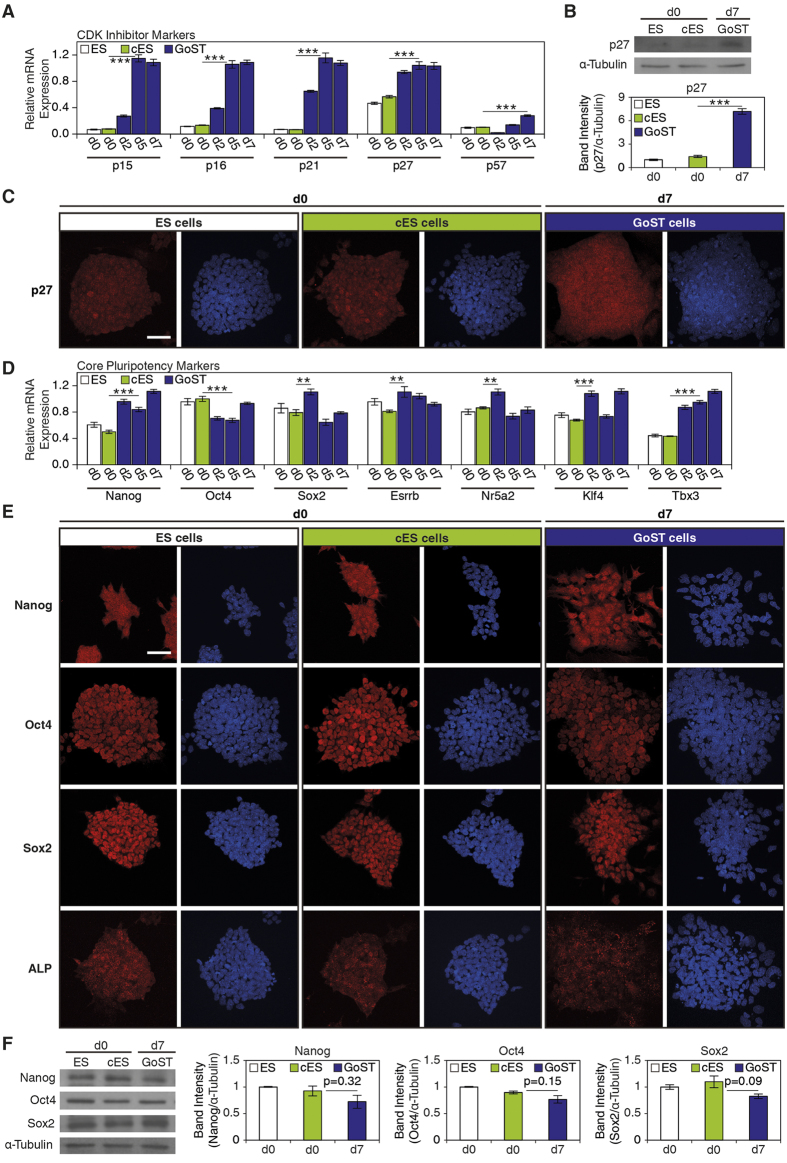
GoST cells upregulate expression of p27, but retain expression of core pluripotency markers compared to ES cells. (**A**) Real-Time PCR analysis of CDK inhibitor markers. Upon GoST induction, expression of *p15*, *p16*, *p21* and *p27* was increased. (**B**) Western blot analysis of p27. Upon GoST induction, expression of p27 was increased. (**C**) Immunofluorescence of p27 (red). DNA was counterstained with DAPI (blue). Upon GoST induction, expression of p27 was increased within cells growing in colonies. Scalebar = 50 μm. (**D**) Real-Time PCR analysis of core pluripotency markers. Upon GoST induction, expression of core pluripotency markers remained mostly unchanged while expression of *Nanog* and *Tbx3* was increased. (**E**) Immunofluorescence of Nanog, Oct4, Sox2 and ALP (red). DNA was counterstained with DAPI (blue). Upon GoST induction, cells continued to express Nanog, Oct4, Sox2 and ALP. Scalebar = 50 μm. (**F**) Western blot analysis of Nanog, Oct4 and Sox2. Upon GoST induction, expression of Nanog, Oct4 and Sox2 showed only a slight decrease and remained at similar levels. Real-Time PCR data were normalized to *Gapdh* and generated from duplicates of two independent experiments. Western blot data were normalized to α-Tubulin and generated from three independent experiments. Error bars correspond to S.E.M. Two stars represent p < 0.01 and three stars represent p < 0.001. See Figure S3.

Analysis of the gene expression of CDK inhibitors revealed that expression of *p15*, *p16*, *p21* and *p27* was gradually increased, while the expression of *p57* did not show a clear increase upon GoST induction (Fig. 3A). p27 is specific to the G1/S phase and is involved in the earliest steps of the mitotic arrest during gonocyte induction at E12.5–13.5^17^ (Fig. 1A). Quantification of p27 expression levels revealed an increase upon GoST induction (Fig. 3B). Immunofluorescence also showed that in GoST cell cultures the expression of p27 was increased in cells located within the colonies compared to ES cells and cES cells (Fig. 3C). Altogether, these results suggest that p27 is involved in promoting the G1/S phase arrest in GoST cells.

### GoST cells retain the expression of core pluripotency markers, thus excluding the possibility of somatic differentiation

Previous studies reported that increased expression of p27 in ES cells correlated with a loss of pluripotency markers^29^. Consequently, we tested for the expression of pluripotency markers and showed that they remained at similar levels (*Oct4*, *Sox2*, *Esrrb*, *Nr5a2* and *Klf4*) or were increased (*Nanog* and *Tbx3*) (Fig. 3D). In addition, immunofluorescence revealed that Nanog, Oct4, Sox2 and alkaline phosphatase (ALP) were still expressed in GoST cells (Fig. 3E). Nanog, Oct4 and Sox2 expression was also quantified, confirming that all three core pluripotency markers remained at similar levels upon GoST induction (Fig. 3F). Altogether, the finding of high expression levels of core pluripotency markers concomitant with an upregulation of p27 and a G1/S phase arrest is remarkable, and according to our knowledge, has never been reported before *in vitro*.

Since LIF was removed from the culture medium during GoST induction, we expected a decrease in the LIF downstream target Stat3 signaling^30^. Western blot analysis confirmed that the ratio of phosphorylated over total Stat3 (P-Stat3/T-Stat3) was decreased upon GoST induction (Fig. S3). Decrease of P-Stat3/T-Stat3 together with increased p27 expression implied that naive pluripotency as it appears in ES cells could not be assumed. The expression of core pluripotency markers implied that GoST cells had not differentiated into a somatic fate.

### Dazl expression levels discriminate two distinct cellular populations

**Figure 4:**
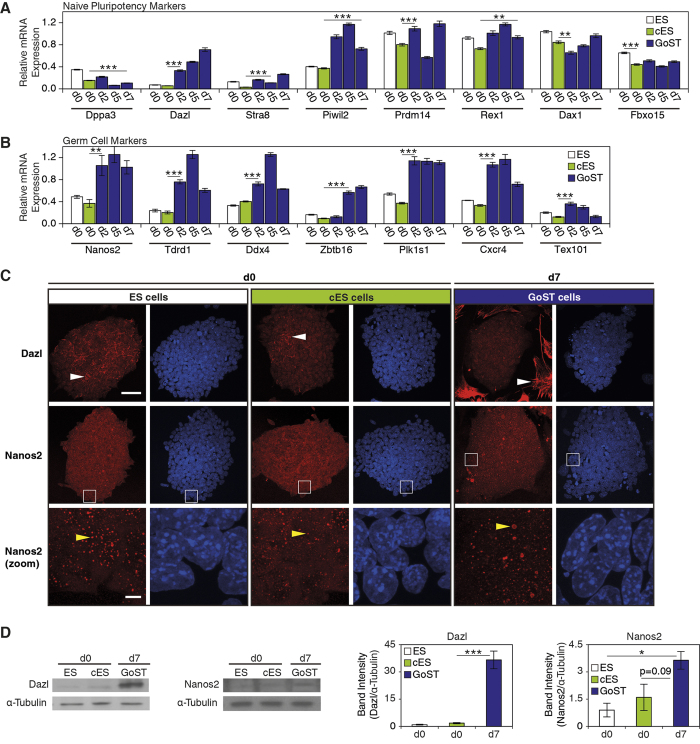
The expression of germ cell-specific genes is modulated in GoST cells, indicative of gonocyte induction. (**A**) Real-Time PCR analysis of naive pluripotency markers. Upon GoST induction, expression of *Dazl* and *Piwil2* was increased, while expression of all other markers remained unchanged. (**B**) Real-Time PCR analysis of germ cell markers. Upon GoST induction, expression of germ cell markers increased except for expression of *Tex101*, which remained unchanged. (**C**) Immunofluorescence of Dazl and Nanos2 (red). DNA was counterstained with DAPI (blue). ES cells and cES cells showed a nuclear and cytoplasmic expression of Dazl within cell colonies, while some cells within the colonies expressed Dazl very strongly in the cytoplasm. Upon GoST induction, all cells located within multicellular colonies continued to express Dazl at the same level as ES cells with low Dazl expression in the nucleus and cytoplasm, while very large cells with strongly increased expression of cytoplasmic Dazl appeared around the cell colonies. White arrowheads indicate cells with strong cytoplasmic Dazl expression. Nanos2 was expressed in ES cells, cES cells and GoST cells and appeared in the form of cytoplasmic round bodies as shown in the zoom images (from white squares). The size of Nanos2 bodies was decreased in cES cells compared to ES cells, but increased in GoST cells compared to ES cells. Yellow arrowheads indicate Nanos2 bodies. Scalebar = 50 μm for upper Dazl and Nanos2 images and scalebar = 5 μm for zoom images. (**D**) Western blot analysis of Dazl and Nanos2. Upon GoST induction, expression of Dazl and Nanos2 was increased. Real-Time PCR data were normalized to *Gapdh* and generated from duplicates of two independent experiments. Western blot data were normalized to α-Tubulin and generated from three independent experiments. Error bars correspond to S.E.M. One star represents p < 0.05, two stars represent p < 0.01 and three stars represent p < 0.001. See Fig. S4.

The expression of genes specific to naive pluripotency^31^ remained at similar levels between ES cells and cES cells for all naive pluripotency genes tested (Fig. 4A). Upon GoST induction, expression of most tested genes remained at similar levels, including *Dppa3*, *Stra8*, *Prdm14*, *Rex1*, *Dax1* and *Fbxo15* (Fig. 4A). However, expression of *Piwil2* and *Dazl* were increased (Fig. 4A). *Piwil2* was reported to be specifically upregulated during male gonocyte induction between E12.5 and E14.5^32^ and its increased expression suggested the presence of germ cell-specific processes.

Dazl was reported to be strongly upregulated during meiosis^33^ and not during gonocyte induction, although its expression at a low level was reported to be essential for gonocyte induction^11^. Western blot analysis showed that Dazl was strongly increased upon GoST induction (Fig. 4D). Immunofluorescence showed expression of Dazl in all ES cells and cES cells, while some cells within the colonies expressed Dazl strongly in the cytoplasm (Fig. 4C, white arrowheads). Interestingly, upon GoST induction, all cells located within the colonies expressed Dazl at a low level similar to ES cells, while much larger cells with strongly increased expression of Dazl in the cytoplasm appeared around the colonies (Fig. 4C). These results implied that GoST cells show low Dazl expression and become physically separated from highly enlarged cells which show high Dazl expression. The strikingly large and flat morphology of cells with high Dazl expression suggested a senescent-like phenotype. Our results suggested a non-meiotic function of Dazl in GoST cells.

### Genes specific to gonocyte induction are upregulated upon GoST induction

We next analyzed expression of genes specific to primed pluripotency, typically expressed by epiblast stem cells^1^ (Fig. S4). Expression of *Nodal* and *Eomes* was increased during the entire course of GoST induction, while expression of *Gata6*, *Foxa2*, *Cer1*, *Sox17*, *T* and *Fgf5* showed a delayed increase. The increased expression of *Nodal*^34^ and *Eomes*^35^ was in agreement with the possibility of gonocyte induction (Fig. S4), while the role of the other primed pluripotency markers remains unclear due to the lack of scientific literature studying these genes in gonocytes. It was also possible that some of the germ layer markers were not associated with GoST cells, but were expressed in other cell populations present in the GoST cell culture. Next, we analyzed expression of genes more specific to gonocyte induction at E12.5–13.5^15^,^36^,^37^ (Fig. 4B). Cell surface expression of Cxcr4 is downregulated in the first postmigratory gonocytes from E13.5 onwards^23^. Expression of *Tex101* marks gonocytes from E14–16 onwards, but is not expressed earlier^24^ (Fig. 1A). Expression was similar between ES cells and cES cells for all genes tested (Fig. 4B). However, expression of all tested genes specific for gonocyte induction at E12.5–13.5 (*Nanos2*, *Tdrd1*, *Ddx4*, *Zbtb16* and *Plk1s1*) was increased upon GoST induction (Fig. 4B). We also measured increased expression of *Cxcr4* (Fig. 4B). However, it was shown in another study that in bone marrow-derived mononuclear cells expression of Cxcr4 was posttranscriptionally controlled by microRNAs^38^. Changes in *Cxcr4* expression may therefore not correlate with its protein expression. As expected, expression of *Tex101* did not show a clear increase upon GoST induction (Fig. 4B). These results support the view that GoST induction *in vitro* produces some molecular markers of gonocyte induction within the male gonads at E12.5–13.5.

### Nanos2 expression is increased in GoST cells

As Nanos2 is an essential driver of male gonocyte induction, and its mRNA levels were increased upon GoST induction, we further investigated the expression of Nanos2 (Fig. 4C,D). Western blot analysis showed that expression of Nanos2 was increased upon GoST induction (Fig. 4D). Immunofluorescence revealed that Nanos2 was expressed in the form of round bodies within ES, cES and GoST cells (Fig. 4C). Interestingly, some of the Nanos2 bodies in GoST cells were much larger than those in ES cells and cES cells (Fig. 4C, zoom images, yellow arrowheads), suggesting that Nanos2 activity was increased in GoST cells. It was reported that increased activity of Nanos2 correlated with an increased number and size of P-bodies to which Nanos2 was located^36^. The upregulated expression and activity of Nanos2 is a very specific and essential molecular marker of male gonocyte induction at E12.5–13.5^15^ (Fig. 1A). To the best of our knowledge, this is the first time that upregulation of Nanos2 could be stimulated in mouse ES cells *in vitro* without genetic manipulation.

### GoST cells develop into Tex101-expressing cells after release from GoST induction

Next, we asked whether GoST cells would differentiate into cells with molecular markers of gonocytes, defined here as GoST gonocyte-like (GoST-GL) cells, when the culture conditions which induce GoST are removed. We looked for expression of Tex101, a marker for gonocytes from E14-16 onwards (Fig. 1A).

**Figure 5:**
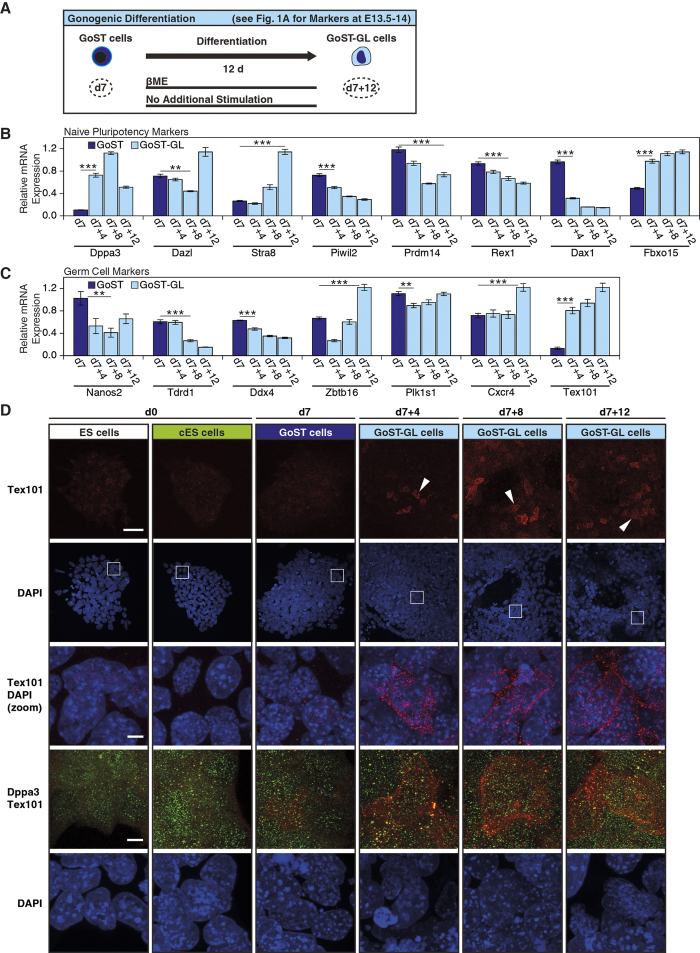
Emergence of cells expressing the gonocyte-specific marker Tex101 after release from GoST induction. (**A**) A schematic representation of the release from GoST induction, leading to a gonogenic differentiation of GoST cells (dark blue) into GoST-GL cells (light blue) with molecular markers of gonocytes at E13.5–14 (see Fig. 1A for markers). GoST cells were cultured in the presence of βME without additional stimulation for 12 days. (**B**) Real-Time PCR analysis of naive pluripotency markers after release from GoST induction. After release from GoST induction, expression of *Dppa3*, *Stra8* and *Fbxo15* was increased, expression of *Dazl* remained unchanged and only was increased on day 12, expression of *Piwil2*, *Prdm14* and *Rex1* was slightly decreased and expression of *Dax1* was strongly decreased. (**C**) Real-Time PCR analysis of germ cell markers after release from GoST induction. After release from GoST induction, expression of *Tex101* was increased, expression of *Cxcr4* remained unchanged and only was increased on day 12, expression of *Zbtb16* was first decreased and increased again on day 12, expression of *Plk1s1* and *Ddx4* remained unchanged and expression of *Nanos2* and *Tdrd1* was decreased. (**D**) Immunofluorescence of Tex101 (red, white arrowheads) and Dppa3 (green) after release from GoST induction. DNA was counterstained with DAPI (blue). Cells expressing Tex101 were detected at 4, 8 and 12 days after release from GoST induction, but could not be detected in ES cells, cES cells or GoST cells. Zoom images (from white squares) showed that Tex101 was associated with the cell membrane. The simultaneous detection of Tex101 and Dppa3 showed that Dppa3 was expressed in ES cells, cES cells, GoST cells and in GoST-GL cells expressing Tex101. Scalebar = 50 μm for upper Tex101 images and scalebar = 5 μm for Tex101 zoom images and Tex101-Dppa3 simultaneous detection images. Real-Time PCR data were normalized to *Gapdh* and generated from duplicates of two independent experiments. Error bars correspond to S.E.M. Two stars represent p < 0.01 and three stars represent p < 0.001. Day 7 data served as reference and is identical to that of charts of GoST induction (Fig. 4). See Figure S5.

For this, we cultured GoST cells for a longer period of time (12 days) in the presence of βME only (Fig. 5A). We then analyzed the expression of all genes previously tested during GoST induction and the most notable findings were as follows: immediately increased *Tex101* and *Dppa3* expression (Fig. 5B,C), decreased *Nanog*, *Oct4*, *Sox2*, *Esrrb*, *Nr5a2*, *Klf4* and *Tbx3* expression (Fig. S5), increased *p27*, *p57* and *Zbtb16* expression on day 12 (Fig. S5 and 5C) and increased *Stra8* and *Dazl* expression on day 12 (Fig. 5B). Increased expression of *Tex101* and *Dppa3* implied the emergence of cells with molecular markers of gonocytes after E14^24^,^39^. This view is supported by decreased expression of *Nanog*, *Oct4*, *Sox2*, *Esrrb*, *Nr5a2*, *Klf4* and *Tbx3* since core pluripotency markers are downregulated during gonocyte maturation after E14^40^. Increased expression of *p27*, *p57* and *Zbtb16* on day 12 also supported this view since *p27* and *Zbtb16* continue to be expressed in gonocytes after E13.5, while *p57* is upregulated from E14.5 onwards^17^. Increased expression of *Stra8* and *Dazl* on day 12, both of which are meiotic genes, implied the emergence of meiotic cells at this late time point. This also agreed with the theory of gonocyte induction since it is known that during prespermatogenesis a fraction of the developing gonocytes undergo meiotic differentiation and apoptosis^41^.

Strikingly, analysis of Tex101 and Dppa3 expression by immunofluorescence detected some cells expressing Tex101 on 4, 8, and 12 days after release from GoST induction (Fig. 5D, white arrowheads) while most cells remained Tex101-negative. Tex101 was associated with the cell membrane in Tex101-expressing GoST-GL cells (Fig. 5D, zoom images), confirming that the subcellular localization is physiological^42^. Furthermore, Dppa3 was detected in these GoST-GL cells as it was in GoST, cES and ES cells (Fig. 5D). Importantly, Dppa3 was detected in all cells expressing Tex101. In contrast to *Tex101*, *Dppa3* is not expressed in spermatocytes^39^, which excluded the possibility that Tex101-expressing GoST-GL cells resembled spermatocytes. To the best of our knowledge, this is the first time that Tex101-expressing cells with some markers of early gonocytes have been created from mouse ES cells *in vitro*.

### GoST cells downregulate Cxcr4 and SSEA1 expression, but continue to express TRA98 after release from GoST induction

Due to the fact that Tex101 was only expressed in some cells, we hypothesized that most Tex101-negative cells would show some molecular markers of gonocytes from an earlier stage than E14. Downregulation of Cxcr4 from E13.5 onwards represents a very early molecular marker of postmigratory gonocytes^23^, while downregulation of SSEA1 from E12.5–15 onwards represents another molecular marker during early gonocyte development with a less well defined time window^20^,^21^ (Fig. 1A). Furthermore, TRA98 is expressed by naive PS cells and the germline including PGCs and gonocytes^7^,^12^,^13^, but not by somatic cells. To the best of our knowledge TRA98 has also not been reported to be expressed in cells of the postimplantation epiblast. Therefore, TRA98 represents a very specific marker to detect a broad range of germ cell types (Fig. 1A).

**Figure 6:**
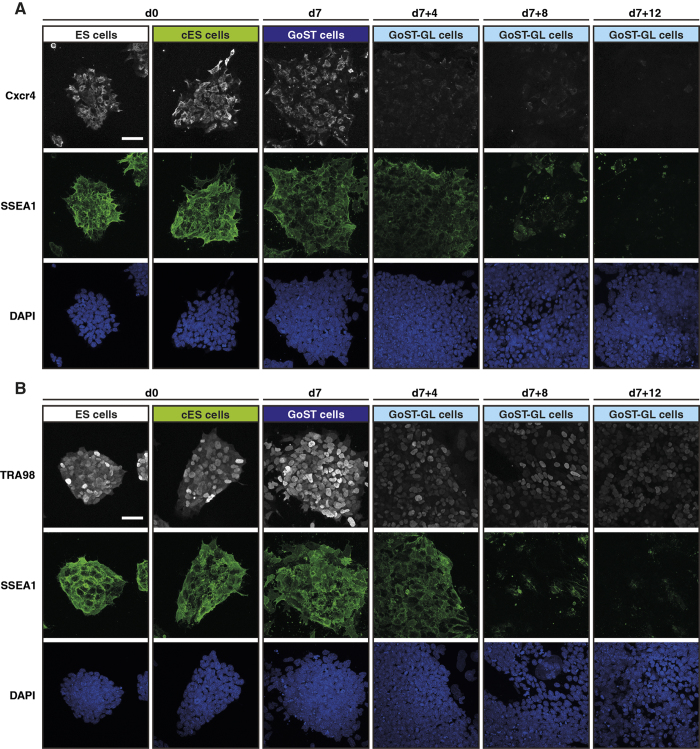
Downregulation of Cxcr4, SSEA1 and expression of TRA98 after release from GoST induction. (**A**) Immunofluorescence of Cxcr4 (white) and SSEA1 (green) after release from GoST induction. Cxcr4 and SSEA1 were detected simultaneously. DNA was counterstained with DAPI (blue). Cxcr4 and SSEA1 were expressed in ES, cES and GoST cells. Cxcr4 was downregulated at 4, 8 and 12 days after release from GoST induction, while SSEA1 remained expressed until 4 days after release from GoST induction and was downregulated afterwards at 8 and 12 days after release. Scalebar = 50 μm. (**B**) Immunofluorescence of TRA98 (white) and SSEA1 (green) after release from GoST induction. TRA98 and SSEA1 were detected simultaneously. DNA was counterstained with DAPI (blue). TRA98 and SSEA1 were expressed in ES, cES and GoST cells. Expression of TRA98 slightly decreased but remained detectable in the nucleus at 4, 8 and 12 days after release from GoST induction, while SSEA1 remained expressed until 4 days after release from GoST induction and was downregulated afterwards at 8 and 12 days after release. Scalebar = 50 μm. See Fig. S6.

Simultaneous detection of Cxcr4 and SSEA1 by immunofluorescence confirmed this hypothesis. While expression of Cxcr4 was high in ES and cES cells, it appeared more heterogeneously distributed in GoST cells (Fig. 6A). Importantly, Cxcr4 expression was downregulated in the majority of cells on 4, 8 and 12 days after release from GoST induction (Fig. 6A). SSEA1 expression was high in ES, cES and GoST cells, as well as in most cells on 4 days, but decreased on 8 and 12 days after release (Fig. 6A).

Expression of TRA98 was detected in ES, cES and GoST cells (Fig. 6B). Although expression of TRA98 was weaker on 4, 8 and 12 days after release from GoST induction, it was still detectable in the nucleus of most cells (Fig. 6B). The simultaneous detection of SSEA1 clearly showed that TRA98 remained expressed while SSEA1 was downregulated in the same cells on 8 and 12 days after the release (Fig. 6B). In a control experiment, SSEA1 and TRA98 were undetectable in mouse embryonic fibroblasts (MEFs), while ES cells expressed both markers (Fig. S6). However, Cxcr4 was detected in both ES cells and MEFs in our control experiment (Fig. S6), which is confirmed in the literature^43^. The broad expression of TRA98 after release from GoST induction clearly revealed a germ cell identity, excluding primed pluripotency and somatic differentiation. Downregulation of SSEA1 in the same cells excluded naive pluripotency. Altogether, the downregulation of SSEA1 in TRA98-expressing cells and the earlier downregulation of Cxcr4 suggest a molecular marker pattern typical for gonocyte induction (Fig. 1A).

### GoST cells exhibit an equal or better *in vitro* multilineage differentiation potential compared to ES cells

**Figure 7:**
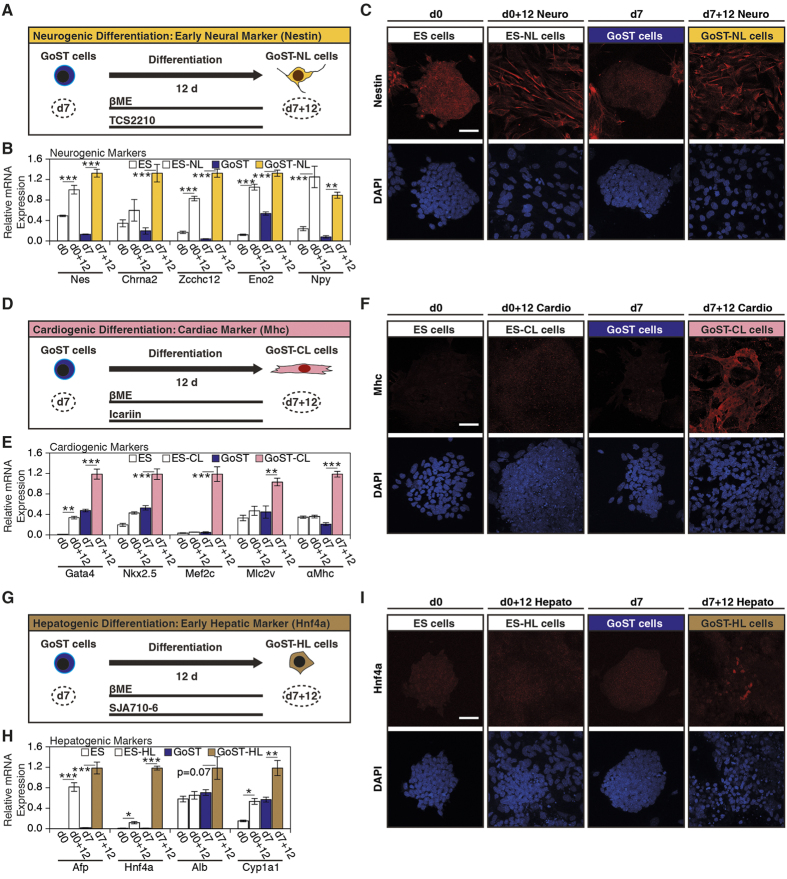
GoST cells exhibit an equal or better *in vitro* multilineage differentiation potential compared to ES cells. (**A**) A schematic representation of the neurogenic differentiation of GoST cells (dark blue) and ES cells (white) into GoST-NL cells (orange) and ES-NL cells (white) for 12 days, respectively. (**B**) Real-Time PCR analysis of neurogenic markers. Expression of *Nes*, *Chrna2*, *Zcchc12*, *Eno2* and *Npy* was increased in GoST-NL and ES-NL cells. (**C**) Immunofluorescence of Nestin (red). Cells expressing Nestin were detected in GoST-NL cells and ES-NL cells. Undifferentiated ES cells (day 0) already showed a strong staining for Nestin, while undifferentiated GoST cells growing in colonies (day 7) showed no Nestin signal. Some large cells surrounding GoST cell colonies stained positive for Nestin. Scalebar = 50 μm. (**D**) A schematic representation of the cardiogenic differentiation of GoST cells (dark blue) and ES cells (white) into GoST-CL cells (pink) and ES-CL cells (white) for 12 days, respectively. (**E**) Real-Time PCR analysis of cardiogenic markers. Expression of *Gata4*, *Nkx2.5*, *Mef2c*, *Mlc2v* and *αMhc* was increased in GoST-CL cells, but only expression of *Gata4* and *Nkx2.5* was increased in ES-CL cells. (**F**) Immunofluorescence of cardiac Mhc (red). Cells expressing Mhc were detected in GoST-CL cells, but not in ES-CL cells. Scalebar = 50 μm. (**G**) A schematic representation of the hepatogenic differentiation of GoST cells (dark blue) and ES cells (white) into GoST-HL cells (brown) and ES-HL cells (white) for 12 days, respectively. (**H**) Real-Time PCR analysis of hepatogenic markers. Expression of *Afp*, *Hnf4a*, *Alb* and *Cyp1a1* was increased in GoST-HL cells, but only expression of *Afp*, *Cyp1a1* and to a lesser degree *Hnf4a* was increased in ES-HL cells. (**I**) Immunofluorescence of Hnf4a (red). Cells expressing Hnf4a localized to cell nuclei were detected in GoST-HL cells, but not in ES-HL cells. Scalebar = 50 μm. DNA was counterstained with DAPI (blue). Real-Time PCR data were normalized to *Gapdh* and generated from duplicates of two independent experiments. Error bars correspond to S.E.M. One star represents p < 0.05, two stars represent p < 0.01 and three stars represent p < 0.001. See Fig. S7.

We then asked how the *in vitro* multilineage differentiation potential of GoST cells compared to that of ES cells. We tested the neurogenic, cardiogenic and hepatogenic differentiation potential of GoST cells compared to ES cells using the chemical inducers TCS2210^44^, Icariin^45^ and SJA710-6^46^, respectively (Fig. 7A,D,G). We described the resulting cells as GoST neurogenic-like (GoST-NL) cells and ES-NL cells, GoST cardiogenic-like (GoST-CL) cells and ES-CL cells, and GoST hepatogenic-like (GoST-HL) cells and ES-HL cells (Fig. 7A,D,G).

Upon neurogenic differentiation, expression of neurogenic markers (*Nes*, *Chrna2*, *Zcchc12*, *Eno2* and *Npy*) was increased in both GoST-NL and ES-NL cells to a similar degree (Fig. 7B). Immunofluorescence of the early neural differentiation marker Nestin showed that it was increased in GoST-NL and ES-NL cells (Fig. 7C). These results suggested that GoST cells and ES cells had a similar potential to differentiate towards a neurogenic lineage. Interestingly, the expression of *Nes*, *Chrna2*, *Zcchc12* and *Npy* was lower in undifferentiated GoST cells compared to undifferentiated ES cells (Fig. 7B). Nestin was moderately expressed in undifferentiated ES cells, but its expression was much weaker in undifferentiated GoST cells (Fig. 7C). It seemed that during GoST induction neurogenic differentiation was strongly inhibited, even though it could be later stimulated by chemical induction.

Upon cardiogenic differentiation, expression of all cardiogenic markers (*Gata4*, *Nkx2.5*, *Mef2c*, *Mlc2v* and *αMhc*) was increased in GoST-CL cells, but only expression of *Gata4* and *Nkx2.5* was increased in ES-CL cells (Fig. 7E). Additionally, immunofluorescence showed that cardiac myosin heavy chain (Mhc) was detectable in GoST-CL cells, but not in ES-CL cells (Fig. 7F). Furthermore, spontaneously beating cell clusters were detectable in GoST-CL cells as early as 8 days after cardiogenic differentiation, while no beating cells were detectable in ES-CL cells at any time point (Mov. S1). These results implied that GoST cells had an enhanced differentiation potential towards cardiogenic lineages compared to ES cells.

Upon hepatogenic differentiation, expression of early hepatogenic markers (*Afp*, *Hnf4a*, *Alb* and *Cyp1a1*) was increased in GoST-HL cells, while only expression of *Afp* and *Cyp1a1* was similarly increased in ES-HL cells (Fig. 7H). Immunofluorescence showed that the early hepatogenic marker Hnf4a was expressed in some GoST-HL cells and localized to cell nuclei, but was not expressed in ES-HL cells (Figs 7I and S7). These results implied that GoST cells had an enhanced differentiation potential towards early hepatogenic lineages compared to ES cells.

In summary, when compared to ES cells *in vitro*, GoST cells showed an equal neurogenic differentiation potential, while their cardiogenic and hepatogenic differentiation potential was enhanced.

## Discussion

In this work we discovered that some important markers of gonocyte induction can be stimulated in mouse ES cells. We achieved this using a chemical treatment, described here as GoST induction (Fig. 1B). The analyzed cellular properties and markers suggest that GoST cells were not in a state of naive pluripotency^31^. This was supported by our findings that GoST cells survived in the absence of βME (Fig. 2A,B), exhibited reduced proliferation with a G1/S phase arrest (Fig. 2C–E), downregulated Stat3 signaling (Fig. S3), upregulated the expression of p27 (Fig. 3A–C) and showed activation of Nrf2 target genes (Fig. S2). A state of primed pluripotency was also not supported by the analyzed markers, since GoST cells retained the expression of most naive pluripotency markers^31^ such as *Prdm14*, *Dazl*, *Dppa3*, *Piwil2*, *Fbxo15*, *Rex1* and *Dax1* (Fig. 4A,C and D) and expressed Nanos2 and Dazl (Fig. 4C). Retained expression of core pluripotency markers in GoST cells did not support the case for somatic differentiation^1^ (Fig. 3D–F). Although these results suggest the absence of naive or primed pluripotency, GoST cells could still be pluripotent per se, as the important core pluripotency markers Nanog, Oct4 and Sox2 remained expressed in GoST cells (Fig. 3E,F). In addition, we could not exclude the possibility that a subpopulation of cells with commitment towards germ-layer differentiation had emerged during GoST induction, indicated by the upregulated expression of genes involved in germ layer formation^1^ such as *Gata6*, *Foxa2*, *Sox17*, *Cer1*, *T* and *Fgf5* (Fig. S4). It also seems possible that some of the germ layer specific genes were involved in the regulation of gonocyte-specific processes, as *Nodal*^34^ and *Eomes*^35^ were shown to be expressed by gonocytes. Upregulation of *Gata6*, *Foxa2* and *Sox17* after the release from GoST induction (Fig. S5) suggests the appearance of endodermal lineages^31^.

GoST cells showed molecular markers in common with differentiating PGCs at E12.5–13.5 (Fig. 1A). This was supported by the upregulation of p27 (Fig. 3A–C), a G1/S phase arrest (Fig. 2E), increased expression of Nanos2 as well as increased size of intracellular Nanos2 bodies (Fig. 4C,D) and upregulation of genes specific to male gonocyte induction such as *Piwil2*, *Nanos2*, *Tdrd1*, *Ddx4*, *Zbtb16* and *Plk1s1* in GoST cells (Fig. 4B)^16^,^32^,^36^,^37^,^47^,^48^. As inhibition of DNMTs by RG108 was an essential component of GoST induction, it is important to note that a genome wide demethylation process also takes place in PGCs from E8.5-13.5^49^ and is a prerequisite for proper gonocyte induction^8^,^49^. The homogeneously low expression of Dazl in all GoST cells within colonies suggested that meiosis signaling was suppressed (Fig. 4C), while high expression of Dazl was exclusively found in the enlarged cells which may have experienced a meiotic-mitotic crisis that led to cellular senescence (Fig. 4C). This is plausible as meiosis suppression is an essential process during male gonocyte induction^15^.

Furthermore, we detected molecular markers of early gonocytes after longer culture of GoST cells without chemical stimulation (Figs 5 and 6), including a few Tex101-expressing cells (Fig. 5A,C,D) as well as the downregulation of Cxcr4, the later downregulation of SSEA1 together with the continued expression of TRA98 in most cells (Fig. 6A,B). The increased expression of Tex101 was accompanied by an increase in the expression of Dppa3 which was reported to be expressed in gonocytes until E15.5^7^,^25^, but not in spermatocytes^39^. We therefore conclude that the Tex101-expressing GoST-GL cells showed molecular markers of gonocytes from E14–15.5^7^,^24^,^25^. Since Cxcr4 is downregulated in postmigratory gonocytes from E13.5 onwards^23^, we could further narrow down the time window for the molecular marker expression of cells released from GoST induction to E13.5–14 (Fig. 1A). SSEA1 is downregulated from E12.5–15 onwards^20^ but the germ cell-specific nuclear protein TRA98 remains expressed in gonocytes^7^,^12^,^13^ (Fig. 1A). Expression of TRA98 in the same cells in which SSEA1 was downregulated strongly supported this view (Fig. 6B). Possible mechanisms involved in GoST induction include epigenetic changes such as DNA methylation, Nrf2 signaling, ROS signaling and metabolic changes (Fig. S2). While not part of this work, dissection of these potential mechanisms may be an interesting subject for future studies.

Besides molecular markers of gonocyte induction, GoST cells also showed an equal neurogenic (Fig. 7A–C) and enhanced cardiogenic (Fig. 7D–F) and hepatogenic (Fig. 7G–I) differentiation potential compared to ES cells. Demethylation of the genome may be one factor that enhanced the multilineage differentiation potential in GoST cells, since demethylation of developmental genes may have facilitated their activation during differentiation.

In summary, we show for the first time that some important molecular markers of the first developmental sexual differentiation program (gonocyte induction) can be induced in male mouse ES cells *in vitro* (GoST induction), and that the resulting GoST cells have an enhanced multilineage differentiation potential *in vitro* compared to mouse ES cells. Discovery of new methods to better manipulate PS cell differentiation *in vitro* has the potential to be extremely useful to the field of stem cell biology and medicine.

## Materials and Methods

### Basic cell culture

Male mouse ES cells were purchased from Millipore (Nanog GFP Reporter cell line, SCR089). All cells were cultured in tissue culture flasks or 8 well μ-Slides (ibidi, 80826), coated with gelatin (0.1%) (Millipore, ES-006-B). ES cells were passaged using accutase (Life Technologies, A11105-01). Basic medium (BM) consisted of Embryomax DMEM (Millipore, SLM-220-B), supplemented with 15% (v/v) Embryomax fetal bovine serum (FBS) (Millipore, ES-009-C), 1 mM sodium pyruvate (Millipore, TMS-005-C), 0.1 mM non-essential amino acids (Life Technologies, 11140), and 2 mM Glutamax-I Supplement (Life Technologies, 35050). ES cells were cultured in basic growth medium (BGM), consisting of BM supplemented with 0.1 mM βME (Life Technologies, 31350-010), 10 ng/ml LIF (Life Technologies, PMC4054) and 0.5 μg/ml puromycin (Life Technologies, A11138-03). Immortalized MEFs were kindly provided by Reinhard Fässler (Max Planck Institute of Biochemistry, Martinsried, Germany). MEFs were cultured in DMEM (Life Technologies, 21885) supplemented with 10% (v/v) FBS (Biowest, S181H).

### Conditioning

cES cells were obtained by culturing ES cells in conditioning medium (CM) consisting of BGM supplemented with 5 μM Ex527 and 100 μM RG108 (Cayman chemical, 10009798 and 13302, respectively) for 10 cell passages. The cells were passaged 1:15–1:20 every 2 days. Ex527 and RG108 were added from stock solutions in dimethyl sulfoxide (DMSO). The total DMSO concentration in CM and BGM was 0.03% (v/v).

### GoST induction

cES cells were seeded at 1 × 10^4^ cells/cm^2^ and cultured in CM for 2 days. No further passages were performed at this point. CM was then switched to reprogramming medium (RM) consisting of BM supplemented with 5 μM Ex527, 100 μM RG108 and 10 μM tBHQ (Sigma-Aldrich, 112941) and cultured for a period of up to 7 days with medium replacement every 2 days. The total DMSO concentration in the culture medium during the reprogramming was 0.04% (v/v). βME, LIF and puromycin were not present in RM. As a negative control, ES cells cultured in BGM supplemented with 0.03% (v/v) DMSO, were subjected to removal of βME, LIF and puromycin (not subjected to GoST induction). In the end, the medium of ES cells consisted of BM supplemented with 0.04% (v/v) DMSO.

### Gonogenic differentiation

GoST cells were cultured for 4, 8, or 12 days in BM supplemented with 0.1 mM βME. The culture medium was replaced every day.

### Neurogenic, cardiogenic and hepatogenic differentiation

GoST cells and ES cells were cultured for 12 days in BM supplemented with 0.1 mM βME and either 20 μM TCS2210^44^ (Tocris Bioscience, 3877), 0.1 μM Icariin^45^ (Sigma-Aldrich, I1286) or 5 μM SJA710-6^46^ (Millipore, 375110). The culture medium was replaced every day.

### Cell viability analysis

The supernatant of the cell cultures was combined and centrifuged together with the adherent cells, dissociated using accutase. Cell viability was determined using a simultaneous staining of 7-AAD for detection of cell membrane permeability and Annexin V-APC (BD Biosciences, 559925 and 550474, respectively) according to the manufacturer’s protocol and analyzed by fluorescence-activated cell sorting (FACS) using the BD Accuri C6 flow cytometer (BD Biosciences).

### EdU incorporation assay

To measure DNA synthesis, an EdU (5-ethynyl-2′-deoxyuridine) incorporation assay was performed by using the Click-iT^®^ EdU Alexa Fluor^®^ 647 Imaging Kit (Life Technologies, C10340) according to the manufacturer’s protocol. EdU incorporation was analyzed by imaging and flow cytometry. After adding 10 μM EdU to the culture medium for 6 hours, the cells were either fixed with formaldehyde (2.5% w/v) in phosphate-buffered saline (PBS) for 20 minutes or collected for flow cytometry analysis.

For imaging, fixed and permeabilized cells were also stained with 3 μg/ml DAPI (Life Technologies) for 10 minutes at room temperature, mounted using ProLong^®^ Gold Antifade Reagent (Life Technologies) and later stored at 4 °C. Images were acquired with a Leica TCS SP5 confocal microscope (Leica Microsystems) using a 20× HCX PL APO CS 0.70 air objective. Alexa Fluor 647 (EdU incorporation) was excited with a 633 nm laser and DAPI with a 405 nm laser. Images were acquired as z-stacks with a step size of 0.5 μm (pinhole diameter of 60.7 μm, 512 × 512 pixels resolution and 775 × 775 μm field of view) and processed as maximum z-projections.

For flow cytometry analysis, after EdU incorporation cells were also stained with 2 μg/ml DAPI, followed by incubation for 30 minutes at 37 °C. Cells were not washed after this step and kept on ice until flow cytometry analysis. Flow cytometry was performed using the BD FACS Aria III flow cytometer (BD Biosciences).

### Cell cycle analysis

Cell cycle analysis was performed by measuring cellular DNA content using FACS. Cells were dissociated using accutase and washed twice with PBS. 1 × 10^6^ cells/ml cells were resuspended in PBS and supplemented with 70% (v/v) absolute ethanol while gently vortexing at half speed. Cells were then incubated on ice for 15 minutes and subsequently stored at −20 °C until staining for flow cytometry analysis. Fixed cells were washed with PBS and stained by resuspension in PBS containing 2.5 μg/ml 7-AAD, 0.05% Triton X-100, 0.1 mg/ml RNAse A (Roche, 10109142001), followed by incubation for 30 minutes at 37 °C. Cells were not washed after this step and kept on ice until flow cytometry analysis. Flow cytometry was performed using the FACS Calibur flow cytometer (BD Biosciences). The cell cycle phase distribution analysis was performed with the software ModFit LT version 3.3 (Verity Software House) using the Sync Wizard with modeling of equally-spaced trapezoids for S-phase and debris and aggregate modeling.

### Light microscopy

After PBS wash and cell fixation with formaldehyde (2.5% w/v) in PBS for 20 minutes, the samples were imaged using an Axiovert 200 M inverted microscope (Carl Zeiss, Jena, Germany) with a 40× LD Plan-Neofluar 0.6 Ph2 objective.

### Videomicroscopy of beating GoST-CL cells

Videomicroscopy of beating GoST-CL cells was performed on an inverted live cell microscope (Nikon TE2000-E) equipped with an electron multiplying charge-coupled device (EM-CCD) camera (Hamamatsu 9100-02). Videos were acquired using a 10× Plan fluor DLL 0.3 phase contrast objective with 1.5× optical zoom at a resolution of 500 × 500 pixels for a 267.5 × 267.5 μm field of view and a frame time of 24 ms. Videos were processed using the ImageJ software (http://rsbweb.nih.gov/ij/).

### Immunofluorescence

The medium of the cells was removed and the cells were washed two times with PBS. Cells were fixed with formaldehyde (2.5% w/v) in PBS for 20 minutes, permeabilized with PBS containing 0.3% Triton-X 100 for 10 minutes and blocked at room temperature for 1 hour in PBS containing 2–3% albumin fraction V (Applichem, A6588), followed by a standard immunofluorescence protocol. Antibody incubations were performed in PBS containing 2–3% albumin fraction V (applichem) for 1 hour at room temperature or overnight at 4 °C for primary antibodies and at a dilution of 1:200 for 1 hour at room temperature for secondary antibodies. In addition cells were stained with 3 μg/ml DAPI in PBS for 10 minutes at room temperature.

The primary antibodies were anti-p27 Kip1 (1:200, Cell Signaling Technology, 3698), anti-Nanog (1:500, Millipore, ab9220), anti-Oct4 (1:200, Abcam, ab19857), anti-Sox2 (1:200, Millipore, mab4343), anti-ALP (1:100, Developmental Hybridoma Bank, B4-78), anti-Dazl (1:100, Abcam, ab34139), anti-Nanos2 (1:500, Acris Antibodies, AP30582PU-N), anti-Tex101 (1:500, kindly provided by Yoshihiko Araki^24^,^50^,^51^,^52^), anti-Dppa3 Stella (1:200, Abcam, ab19878), anti-Cxcr4 (1:100, R&D Systems, MAB21651), anti-SSEA1 (1:200, Millipore, mab4301), anti-TRA98 (1:200, Abcam, ab82527), anti-Nestin (1:1000, Abcam, ab81755), anti-Heavy chain cardiac myosin (1:200, Abcam, ab15), anti-Hnf4a (1:1000, Abcam, ab41898) or anti-Nrf2 (1:100, Abcam, ab31163).

Cells were mounted using ProLong^®^ Gold Antifade Reagent (Life Technologies) at room temperature overnight. Images were acquired as z-stacks (0.5 μm step size) with a Leica TCS SP5 confocal microscope with a 63× PL APO CS 1.4 oil objective (pinhole diameter of 95.5 μm, 512 × 512 pixels resolution and a 245 × 245 μm field of view) and processed as maximum z-projections.

Alexa Fluor 546 and Alexa Fluor 555 (Life Technologies) were excited with a 561 nm laser, Alexa Fluor 633 with a 633 nm laser and DAPI was excited with a 405 nm laser. Images were processed using the ImageJ software (http://rsbweb.nih.gov/ij/) and Adobe Photoshop (Adobe Systems Inc.).

### Western blot analysis

The whole cell lysate extracts were collected by scraping cells on ice using 250 ul of RIPA buffer (Sigma-Aldrich, R0278) combined with protease and phosphatase inhibitors (Sigma-Aldrich). After collection, the samples were incubated on an end-over-end shaker for 10 minutes at 4 °C. Small aliquots (20 μl) were collected for protein quantification using the Pierce BCA protein assay (Thermo Scientific, 23225). The remaining sample was incubated with 5x Laemmli Sample Buffer (5% β-mercaptoethanol, reducing agent) for 5 minutes at 95 °C and stored at −20 °C. The western blot samples (10 mg/well) were fractionated by SDS-PAGE (10% or 7% polyacrylamide) and transferred to a polyvinylidene difluoride (PVDF, Bio-Rad, 162–0177) membrane, according to the manufacturer’s protocols (Bio-Rad). The membrane incubations were performed in 10× Roti-Block (Carl Roth, A151.1) TBS-T buffer, overnight at 4 °C for primary antibodies and 1 hour at room temperature for secondary antibodies.

The primary antibodies were anti-alpha-tubulin (1:1000, Abcam, ab7750), anti-Cyclin D1 (1:1000, Cell Signaling Technology, 2926), anti-Cyclin B1 (1:1000, Cell Signaling Technology, 4138), anti-p27 Kip1 (1:1000, Cell Signaling Technology, 3698), anti-Nanog (1:1000, Millipore, ab9220), anti-Oct4 (1:500, Millipore, ab3209), anti-Sox2 (1:1000, Millipore, mab4343), anti-Phospho Stat3 (1:1000, Cell Signaling Technology, 9131), anti-total Stat3 (1:1000, Cell Signaling Technology, 9139), anti-Dazl (1:1000, Abcam, ab34139), anti-Nanos2 (1:500, Acris Antibodies, AP30582PU-N).

Blots were further developed following the manufacturer’s protocol using ECL plus substrate (GE Healthcare, RPN2133) and CDPstar substrate (Sigma-Aldrich, C0712), respectively.

### Real-Time PCR analysis

Total RNA was isolated from ~1 × 10^6^ cells per sample using the RNeasy Plus Mini Kit (Qiagen, 74134) with RNase-free DNase treatment (Qiagen, 79254) following the manufacturer’s protocol. For reverse transcription (RT) 2 μg total RNA was reverse transcribed into cDNA in a reaction volume of 20 μl using the iScript™ Advanced cDNA Synthesis Kit (Bio-Rad, 170-8842). Real-Time PCR reactions were performed on the resulting cDNA using the CFX Connect™ Real-Time PCR Detection System (Bio-Rad) and SsoAdvanced™ SYBR^®^ Green Supermix (Bio-Rad, 172–5262).

PCR reactions were incubated at 95 °C for 3 minutes, followed by 39 cycles of 95 °C for 10 s and 62 °C for 30 s; and were run as duplicates of two independent experiments. PCR Primers were designed using PrimerSelect from the Lasergene software suite (DNASTAR). All primers were synthesized at Microsynth. Results were analyzed using CFX Manager™ software (Bio-Rad) and expression levels relative to *Gapdh* were calculated based on the ∆∆Ct method. Gene abbreviations, NCBI reference sequences for mRNA (used as template for primer design), amplicon size and primer sequences are indicated in Table S1.

### Statistical analysis

Statistically significant differences between data values were calculated using one-way ANOVA and the Tukey post hoc test (one star represents p < 0.05, two stars represent p < 0.01 and three stars represent p < 0.001).

## Additional Information

**How to cite this article**: Moshfegh, C. *et al*. A gonogenic stimulated transition of mouse embryonic stem cells with enhanced control of diverse differentiation pathways. *Sci. Rep*. **6**, 25104; doi: 10.1038/srep25104 (2016).

## Supplementary Material

Supplementary Information

Supplementary Video S1
