# Supplementary material for: A gonogenic stimulated transition of mouse embryonic stem cells with enhanced control of diverse differentiation pathways
Source: Sci Rep. 2016 May 9;6:25104. doi: 10.1038/srep25104 (PMC4860597; doi:10.1038/srep25104)
Supplement: Supplementary Information [file srep25104-s1.pdf]

Supplementary Information

Title

A gonogenic stimulated transition of mouse embryonic stem cells with enhanced control of diverse differentiation pathways

Authors

Cameron Moshfegh, Lina Aires, Malgorzata Kisielow and Viola Vogel

Supplementary Results

Rationale of GoST induction

The findings reported here were made in the context of the initial aim to induce a proliferation arrest in mouse ES cells in the absence of leukemia inhibitory factor (LIF) and β-mercaptoethanol (βME) without loss of cell viability for at least several days. While LIF promotes the naive pluripotent state, βME is an external antioxidant that increases the amount of intracellular reduced glutathione protecting the cells from oxidative stress. We removed LIF to prevent interference with pluripotency signaling and βME to prevent interference with reactive oxygen species (ROS) signaling because such a proliferation arrest may involve signaling different from that in ES cells. Upon withdrawal of βME the ES cells died within 5-7 days, most likely due to oxidative stress which is confirmed in the literature[1]. In this context we treated ES cells with different chemicals: chemicals tested to induce a proliferation arrest included the CDK inhibitor 6,7,4'-trihydroxyisoflavone[2], the DNA polymerase inhibitor aphidicolin[3], the DNA methyltransferase (DNMT) inhibitor RG108[4] and the Sirtuin1 (Sirt1) inhibitor Ex527[5]. Chemicals tested to prevent cell death included the synthetic superoxide dismutase/catalase mimetic EUK 134[6], the general caspase inhibitor Q-VD-OPh[7], the Bax inhibiting peptide V5[8], the checkpoint kinase 2 inhibitor Chk2 inhibitor II[9], the inhibitor of mitochondrial p53-binding pifithrin-μ[10], the mitochondrial permeability transition pore inhibitor BBMP[11], the Bax channel blockers iMAC1 and iMAC2[12] and the nuclear factor erythroid 2-related factor 2 (Nrf2) activators Al-1[13] and tert-butylhydroquinone (tBHQ), while tBHQ also is an electrophilic redox-cycling compound[14]. Only a particular combination of Ex527, RG108 and tBHQ was able to induce a proliferation arrest and at the same time prevent cell death in ES cells when LIF and βME were removed (Fig. S1). However, none of these three molecules alone was able to rescue cell viability. All other tested chemicals and combinations thereof also failed to rescue cell viability.

When we investigated the ES cells that had been treated with this particular procedure, we noticed a striking pattern of molecular markers typical for differentiating primordial germ cells (PGCs) (gonocyte induction). Since we used C57/BL6 male (xy) mouse ES cells in this study, we focused our experiments on markers of gonocyte induction in the male gonads[15,16,17,18,19]. We concluded that some molecular markers of gonocyte induction can indeed be stimulated in mouse ES cells using a combined chemical treatment with RG108,

Ex527 and tBHQ. We defined this particular state as gonogenic stimulated transition (GoST) and the resulting cells as GoST cells (Fig. 1B). Gonocyte induction-specific changes including cell cycle checkpoint activation, gene and protein expression and after longer culture without chemical stimulation, specific changes in molecular marker expression typical for early gonocytes (Fig. 1A) implied that GoST cells showed some markers of differentiating PGCs at E12.5-13.5. We further asked whether this chemical treatment would lead to an improved control of the multilineage differentiation potential of the mouse ES cells. Indeed, GoST cells showed an enhanced multilineage differentiation potential compared to ES cells *in vitro*.

Analysis of metabolic markers

tBHQ is also known to increase intracellular ROS which can promote cell death, but may also regulate cell proliferation and differentiation[20,21] (Fig. S2A). Since ROS are generated during aerobic metabolism typically by oxidative phosphorylation, we then analyzed the expression of Nrf2-unrelated genes involved in basic cellular metabolism (Fig. S2E)[22]. Interestingly, expression of some oxidative phosphorylation markers (*Cox6b2*, *Cox8c*, *Sco2*) was increased, while expression of glycolytic markers (*Ldha* and *Pdk1*) was decreased upon GoST induction (Fig. S2E). Expression of *Cox7a1* and *Pygl* remained unchanged, while *Gys1* expression increased during GoST induction (Fig. S2E). These results implied a decreased glycolysis, increased oxidative phosphorylation and glycogen synthesis without an increase in glycogen breakdown upon GoST induction. It is known that PS cells rely more on glycolysis than on oxidative phosphorylation for their energy metabolism, while differentiated somatic cells mainly use oxidative phosphorylation[22]. When PS cells differentiate into somatic cells they switch from glycolysis to oxidative phosphorylation[22]. Our results of decreased glycolysis and increased oxidative phosphorylation markers upon GoST induction suggest a somatic differentiation. There was also an indication that germ cell-specific metabolic processes had appeared since *Cox6b2* was reported to be specifically expressed in the testis[23], while glycogen represents the main source of energy within the seminiferous tubules[24,25].

Supplementary Figures

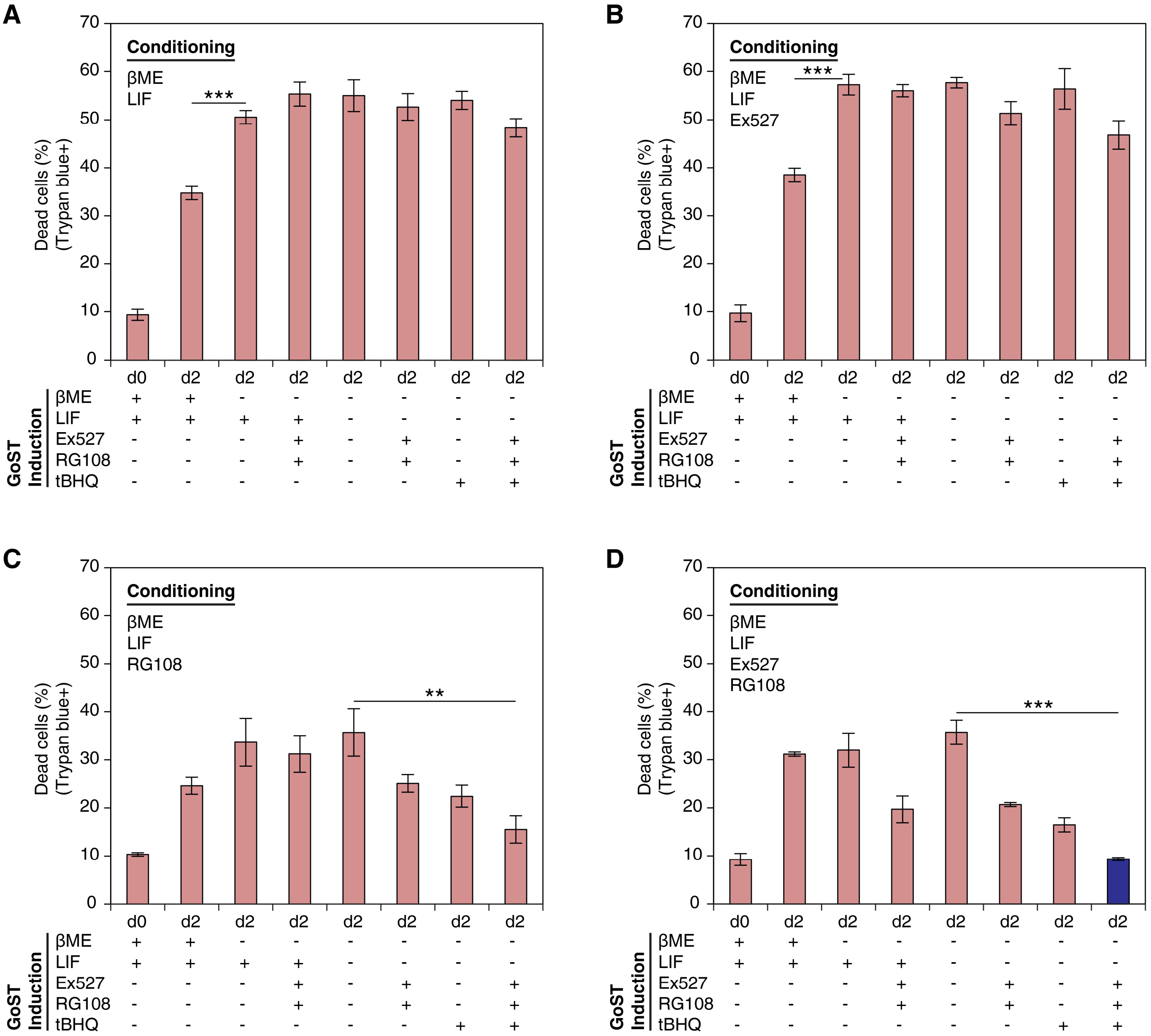

Figure S1, Combinatorial test of the chemical components used for conditioning and GoST induction, Related to Figure 2

Percentage of dead cells for conditioning with different combinations consisting of (A) βME and LIF; (B) βME, LIF and Ex527; (C) βME, LIF and RG108; (D) βME, LIF, Ex527 and RG108 with the respective combinations for GoST induction. The percentage of dead cells was determined by Trypan blue staining and manual counting. The combination which produced the lowest percentage of dead cells is marked by the blue bar in (D). Data were generated from four independent measurements with samples pooled from four independent experiments and error bars correspond to S.E.M. Two stars represent $p < 0.01$ and three stars represent $p < 0.001$.

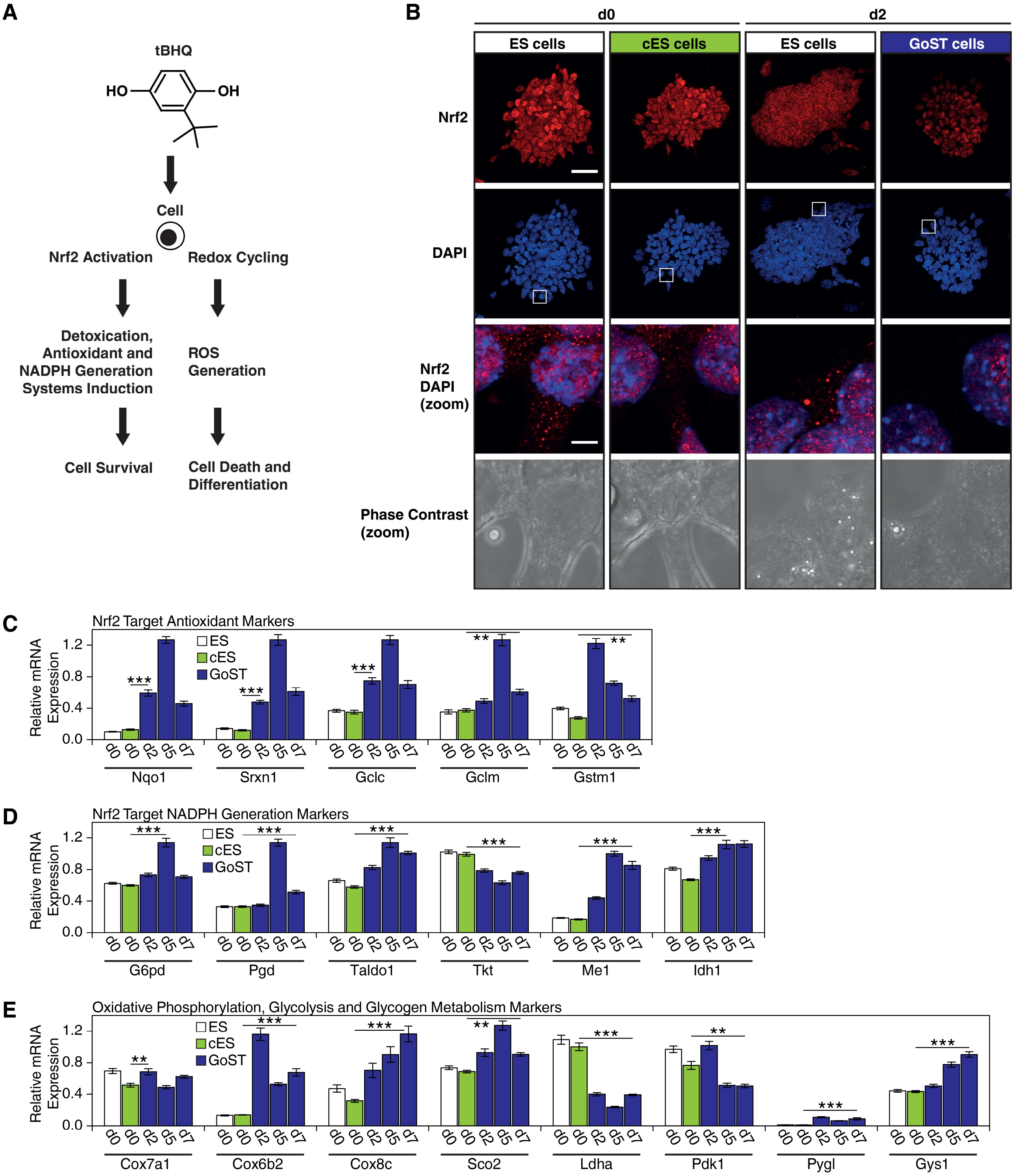

Figure S2, Nrf2 target genes are activated and genes involved in metabolism are differentially modulated upon GoST induction, Related to Figure 2

(A) A schematic representation of tBHQ-induced cellular responses. tBHQ activates Nrf2 which induces the expression of detoxication, antioxidant and NADPH generation systems, contributing to cell survival. tBHQ

also undergoes redox cycling in the cell and causes the generation of ROS which can modulate cell proliferation, differentiation and cell death.

(B) Immunofluorescence of Nrf2 (red). DNA was counterstained with DAPI (blue). Nrf2 was expressed in ES cells, cES cells and GoST cells. Upon 2 days of GoST induction, Nrf2 showed a typical nuclear staining pattern and appeared to be depleted in the cytoplasm of GoST cells (zoom image from white squares). ES cells deprived of LIF and βME for 2 days showed a more diffuse localization of Nrf2 within cells clearly showing strong cytoplasmic localization of Nrf2. Respective, phase contrast zoom images at the bottom show the membrane boundaries of cells. Scalebar = 50 µm for upper Nrf2 and DAPI images and scalebar= 5 µm for zoom images.

(C) Real-Time PCR analysis of Nrf2 target antioxidant markers. Upon GoST induction, expression of all Nrf2 target antioxidant markers was increased.

(D) Real-Time PCR analysis of Nrf2 target NADPH generation markers. Upon GoST induction, expression of *G6pd*, *Pgd*, *Taldo1*, *Me1* and *Idh1* was increased, while expression of *Tkt* was slightly decreased.

(E) Real-Time PCR analysis of oxidative phosphorylation, glycolysis and glycogen metabolism markers. Oxidative phosphorylation markers consisted of *Cox7a1*, *Cox6b2*, *Cox8c* and *Sco2*. Glycolysis markers consisted of *Ldha* and *Pdk1*. Glycogen metabolism markers consisted of *Pygl* and *Gys1*. Upon GoST induction, expression of *Cox6b2*, *Cox8c*, *Sco2* and *Gys1* was increased, expression of *Cox7a1* and *Pygl* remained unchanged and expression of *Ldha* and *Pdk1* was decreased.

Real-Time PCR data were normalized to *Gapdh* and generated from duplicates of two independent experiments. Error bars correspond to S.E.M. Two stars represent $p < 0.01$ and three stars represent $p < 0.001$.

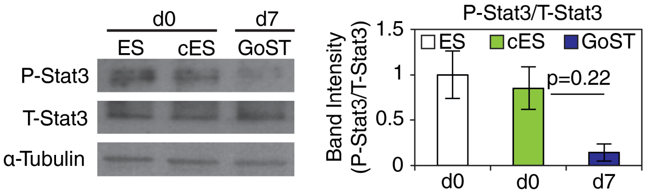

Figure S3, Stat-3 phosphorylation is decreased upon GoST induction, Related to Figure 3

Western blot analysis of P-Stat3 over T-Stat3. Upon GoST induction, the ratio of P-Stat3/T-Stat3 was decreased. Data were generated from three independent experiments and error bars correspond to S.E.M.

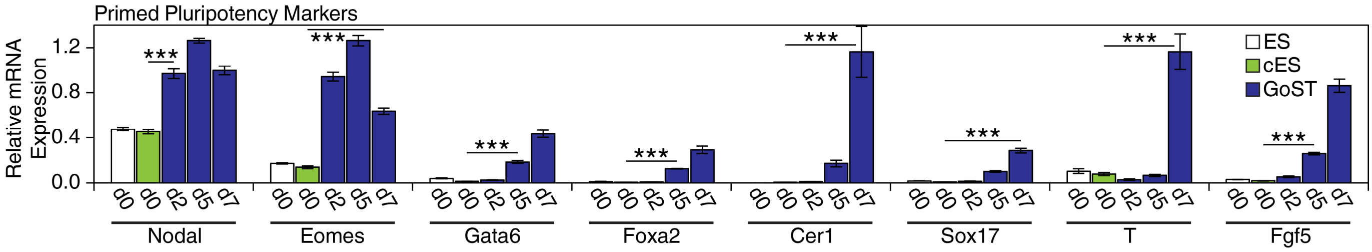

Figure S4, The expression of genes involved in early germ layer formation is increased upon GoST induction, Related to Figure 4

Real-Time PCR analysis of primed pluripotency markers. Upon GoST induction, expression of *Nodal* and *Eomes* was increased, while expression of *Gata6*, *Foxa2*, *Cer1*, *Sox17* and *Fgf5* was increased after day 5 of GoST induction and expression of *T* was increased after day 7 of GoST induction. Data were normalized to *Gapdh* and generated from duplicates of two independent experiments. Error bars correspond to S.E.M. Three stars represent $p < 0.001$.

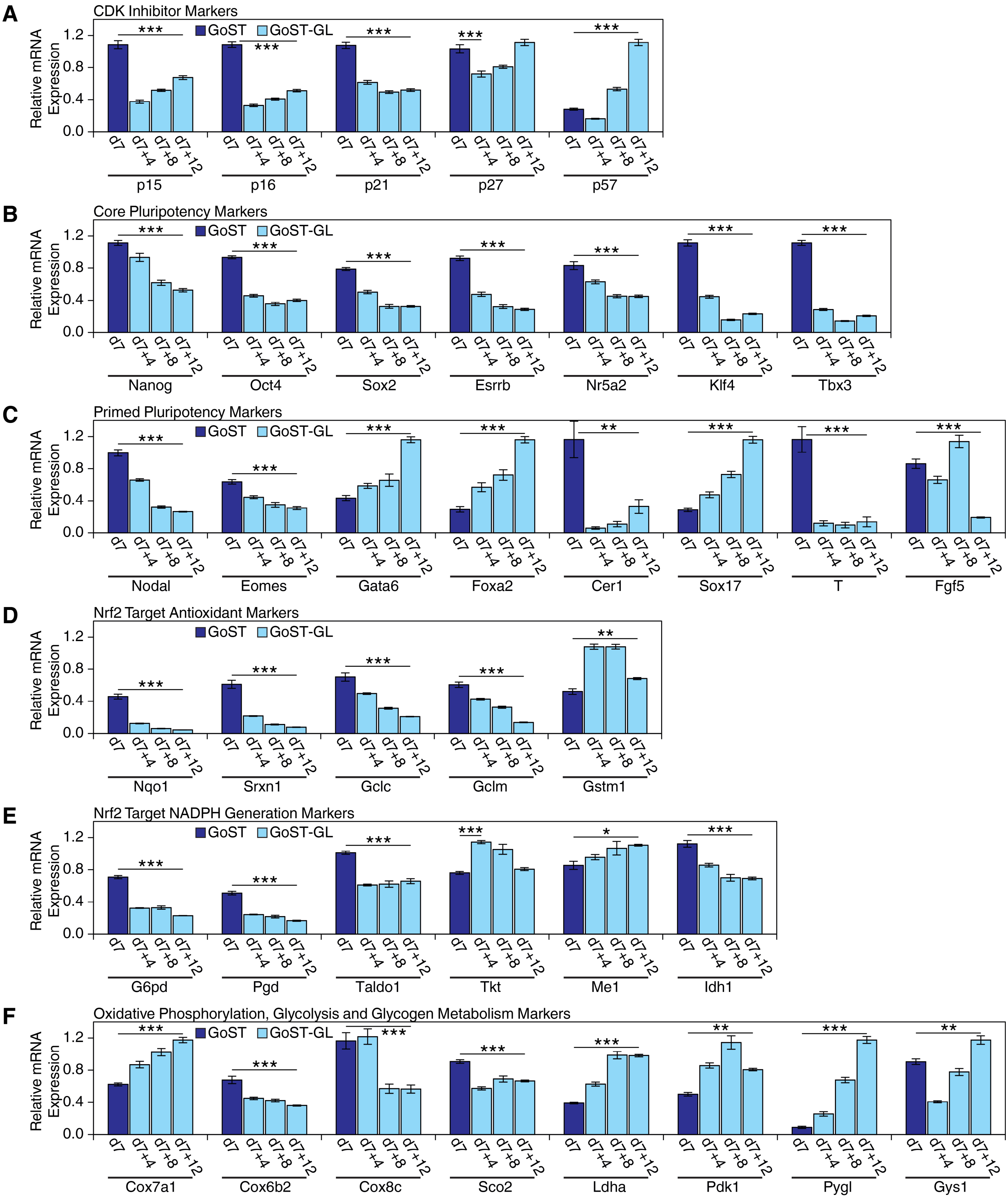

Figure S5, Expression of various genes after release from GoST induction, Related to Figure 5

(A) Real-Time PCR analysis of CDK inhibitor markers after release from GoST induction. After release from GoST induction, expression of *p15*, *p16* and *p21* was decreased, while expression of *p27* and *p57* was

first decreased and increased again on day 12. Day 7 data served as reference and is identical to that of charts of GoST induction (Fig. 3A).

(B) Real-Time PCR analysis of core pluripotency markers after release from GoST induction. After release from GoST induction, expression of core pluripotency markers was decreased and most strongly in *Klf4* and *Tbx3*. Day 7 data served as reference and is identical to that of charts of GoST induction (Fig. 3D).

(C) Real-Time PCR analysis of primed pluripotency markers after release from GoST induction. After release from GoST induction, expression of *Nodal*, *Eomes*, *Cer1*, *T* and *Fgf5* was decreased and expression of *Gata6*, *Foxa2* and *Sox17* was increased. Day 7 data served as reference and is identical to that of charts of GoST induction (Fig. S4).

(D) Real-Time PCR analysis of Nrf2 target antioxidant markers after release from GoST induction. After release from GoST induction, expression of *Nqo1*, *Srxn1*, *Gclc* and *Gclm* was decreased and expression of *Gstm1* was increased. Day 7 data served as reference and is identical to that of charts of GoST induction (Fig. S2C).

(E) Real-Time PCR analysis of Nrf2 target NADPH generation markers after release from GoST induction. After release from GoST induction, expression of *G6pd*, *Pgd*, *Taldo1* and *Idh1* was slightly decreased, while expression of *Me1* and *Tkt* was slightly increased. Day 7 data serves as reference and is identical to that of charts of GoST induction (Fig. S2D).

(F) Real-Time PCR analysis of oxidative phosphorylation, glycolysis and glycogen metabolism markers after release from GoST induction. Oxidative phosphorylation markers consisted of *Cox7a1*, *Cox6b2*, *Cox8c* and *Sco2*. Glycolysis markers consisted of *Ldha* and *Pdk1*. Glycogen metabolism markers consisted of *Pygl* and *Gys1*. After release from GoST induction, expression of *Cox7a1*, *Ldha*, *Pdk1* and *Pygl* was increased, expression of *Gys1* was first decreased and increased again on day 12, expression of *Cox8c* was decreased and expression of *Cox6b2* and *Sco2* was slightly decreased. Day 7 data serves as reference and is identical to that of charts of GoST induction (Fig. S2E).

Real-Time PCR data were normalized to *Gapdh* and generated from duplicates of two independent experiments. Error bars correspond to S.E.M. One star represents $p < 0.05$, two stars represent $p < 0.01$ and three stars represent $p < 0.001$.

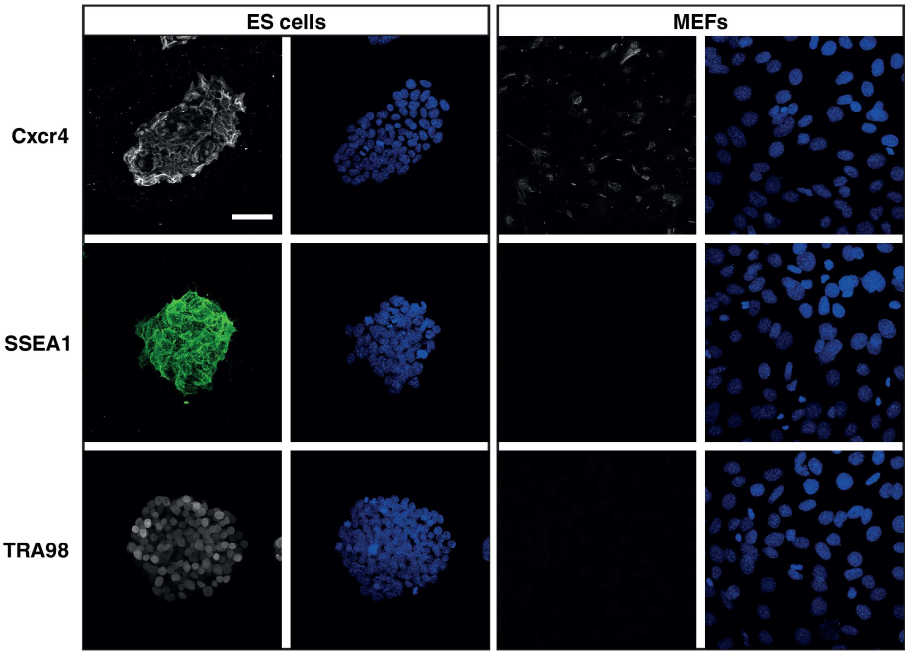

Figure S6, Expression of Cxcr4, SSEA1 and TRA98 in ES cells compared to MEFs, Related to Figure 6

Immunofluorescence of Cxcr4 (white), SSEA1 (green) and TRA98 (white) in ES cells and MEFs. DNA was counterstained with DAPI (blue). Cxcr4, SSEA1 and TRA98 were expressed in ES cells. MEFs expressed Cxcr4, but did not express SSEA1 or TRA98. Scalebar = 50 µm.

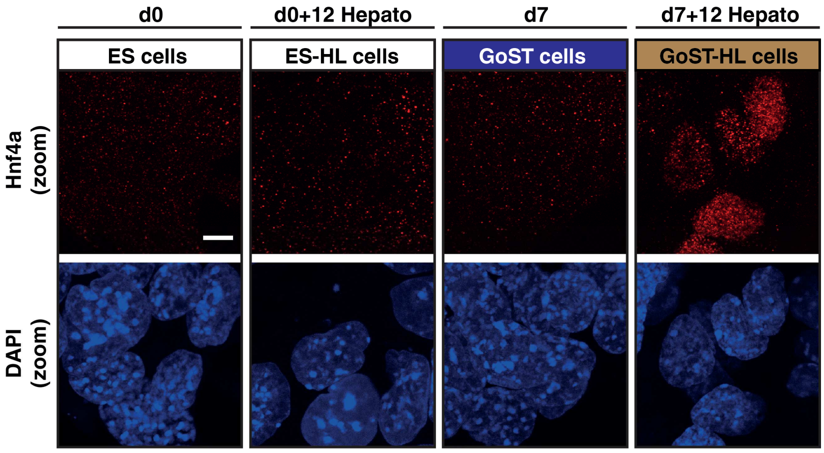

Figure S7, Increased expression of Hnf4a localizes to the cell nucleus upon hepatogenic differentiation of GoST cells, Related to Figure 7

Immunofluorescence zoom images of Hnf4a (red) after hepatogenic differentiation of 12 days. DNA was counterstained with DAPI (blue). After 12 days of hepatogenic differentiation, cells expressing Hnf4a localized to cell nuclei were detected in GoST-HL cells, but not in ES-HL cells. Scalebar = 5µm.

Movie S1, Spontaneously beating cells appear upon cardiogenic differentiation of GoST cells, Related to Figure 7

Real-time movie from the time-lapse video microscopy of beating cells after cardiogenic differentiation of GoST cells. Spontaneously beating cells were observed in GoST-CL cells, which appeared in isolated patches as early as 8 days upon cardiogenic differentiation, while no beating cells were detected in ES-CL cells at any timepoint. Scalebar = 50 µm.

**Gene**	**mRNA ID (NCBI Reference Sequence)**	**Amplicon Size (bp)**	**Forward Primer Sequence (5'→3')**	**Reverse Primer Sequence (5'→3')**
Gapdh	NM_008084	90	ACCCCCAATGTGTCCGTCGTG	AGATGCCTGCTTCACCACCTTCTTG
p15	NM_007670	133	CCGCCTGCCGGTAGACTTGG	CTAGATGGGGCTGGGGAGAAAGAAG
p16	NM_001040654	73	CAAGAGCGGGGACATCAAGACATC	ACCACCCAGCGGAACACAAAGAG
p21	NM_007669	65	AGATCCACAGCGATATCCAGACATTCA	TCGGACATCACCAGGATTGGACAT
p27	NM_009875	141	AAGCACTGCCGGGATATGGAAGAA	GCGCGGGGGCCTGTAGTAGAA
p57	NM_009876	119	CTGAACGCCGAGGACCAGAACC	CGGTAGAAGGCGGGCACAGACT
Nanog	AB093574	101	ACCTCAGCCTCCAGCAGATGCAA	CCGCTTGCACTTCACCCTTTGG
Oct4	NM_013633	135	TGCTGAAGCAGAAGAGGATCACCTTG	TGTTCTTAAGGCTGAGCTGCAAGGC
Sox2	NM_011443.3	139	AGGGTTCTTGCTGGGTTTTGATTCTG	AACGGTCTTGCCAGTACTTGCTCTCAT
Esrrb	NM_011934	262	TGCCCGGGACCCAAGAGACATA	AGTGAGTTCCGGCTGGCTGAGGT
Nr5a2	NM_030676	152	CGCATGGGAAGGAAGGGACAAT	CCGCTGATCGAACTGAAGGGAAC
Klf4	NM_010637	111	GCCATTATTGTGTCGGAGGAAGAGGA	GCTCCCCCGTTTGGTACCTTTAGAAC
Tbx3	NM_011535	127	CGCCCTGTCCCTTTCAGTTTTGTC	AGTCCCGCGTTTCAAAGCAACAG
Nqo1	NM_008706	79	CATCCTGCGTTTCTGTGGCTTCC	GCGGGCATCTGGTGGAGTGTG
Srxn1	NM_029688	113	TTGGCTGCATTGGCTTGGTTACTCT	CGCTCCAGGCCCTCTCACTACTACTC
Gclc	NM_010295	142	TGCGGAGGCATCAAAGGCTTCT	AGTGGCCAGCTGATCATAAAGGTATCTTG
Gclm	NM_008129	200	ATGGCTTCGCCTCCGATTGAA	CAGGAGGCCAGGTTAACTTGGTTACTATT
Gstm1	NM_010358	144	ACGTCCGCGGACTGACACACC	AGTCCAGGCCCAGCTTGAACTTCTC
G6pd	NM_008062	111	CTATGCCCGCTCACGACTCACAGT	GAATTACGGGCAAAGAACTCCTCCAG
Pgd	NM_001081274	146	GAACCGGAGAACCCTGCTGTGACT	ATGCCGCATGCCCAGAACATC
Taldo1	NM_011528	191	GGTGGGCCTCAAGAGGAGCAGAT	TTGCCGACCCCAGCTTCTTTGTA
Tkt	NM_009388	210	GCCACGCCAGTGACCGTATCATT	GGTCGAAGGCCCGTGTGAAGAA
Me1	NM_008615	294	TGCCTTGGGGATTGCTCACTTG	ATGATGGGCCGCTCGTTGAAG
Idh1	NM_001111320	180	CCTGGGCCTGGAAAAGTAGAGATAACC	AGGCCAGCCCTTGGACAGAGC
Cox7a1	NM_009944	93	GAAAGGCGGGGGAATGGACAAC	GCCCAGCCCAAGCAGTATAAGCAGTA

Cox6b2	NM_183405	95	CCTTTGATCCGCGCTTCCCTAAC	GCGGCGATTCATGGTCTTCACAC
Cox8c	NM_001039049	103	GCCGCCTCAGCCACTCAGAAAG	GCAGCTGGGATGTAAAAGGTCGTAAAAA
Sco2	NM_001111288	186	CCGCGAGGGCTGAGAAGGAAC	CGGGGCAAATATCAGGGCAGTG
Ldha	NM_010699	127	AGCGCGGTTCCGTTACCTGATG	GACGCCGGCAACATTCACACC
Pdk1	NM_172665	255	GCTAGGCGGCTTTGTGATTTGTATTATGTTA	TCCCCGGTCACTCATCTTCACAGTC
Pygl	NM_133198	252	CAGACGGGGACAAAGTGGGTCG	CAGCCGCAACTCCTTCCCTTCA
Gys1	NM_030678	241	AAGCCGTGCGCAAACAACTATGG	ATTCGGCGGATGGTGGTCAAGAT
Dppa3	NM_139218	181	TGCAGCCGTACCTGTGGAGAACA	GTCCCGTTCAAACTCATTTCCTTCG
Dazl	NM_010021	262	ACCTCCGGCTTATACAACTGTTAACTACCA	AAGCACTGCCCGACTTCTTCTGAA
Stra8	NM_009292	130	CAGCGCTATGTTTGCCACCTGC	TGGGGGCTCTGGTTCCTGGTT
Piwil2	NM_021308	152	TCCGCAAGGACAGAGAAGAACCC	TGCTCGTCCCAGTGGTAACAGAGAG
Prdm14	NM_001081209	113	CGCCACCACCGAGGAGGAGT	CCGGGTTCAACAAGGGAGCAGT
Rex1	NM_009556	144	TGGCTGCGAGAAGAGCTTTATTCAGTC	CGTGTATCCCCAGTGCCTCTGTCAT
Dax1	NM_007430	115	GCGAGTGGTGGCAGCTGTCCTAC	GGCTGCTCTTCACCGCACACATAG
Fbxo15	NM_015798	125	GTGGGGCTGTGGCAGGAGAATG	GTAGTGTCGGGAGGCAATGTATAGGGAA
Nanos2	NM_194064	76	ACCGGCGACCAGGCTCATACAC	GCCCACTGCGTCGGTAGAGAGACT
Tdrd1	NM_001002238	244	GTGGCCTGGCAGAAAACCTCACTT	CTGGCGTTTGCTGTCTCTTTCTTCC
Ddx4	NM_010029	100	AACGCCAAACCCTTTTATTCAGTGCTAC	TGCCCAACAGCGACAAACAAGTAACT
Zbtb16	NM_001033324	86	CCGCCCATTTTTACCCCCTACAA	ACCCCAGCCCATATCCTCTCAACA
Plk1s1	NM_001033298	132	AGATTCGCTCTTCACGGAATATGTTTTACA	CATTGCCCTCGTGTCTTCAATAAGTGAC
Cxcr4	NM_009911	157	GACCGCCTTTACCCCGATAGCC	TGAGGGCCTTGCGCTTCTGG
Tex101	NM_019981	225	CTAATCGCCTCACGTTGGACTCTGG	CACCGCCTCTCCTCCTTGAGAAAC
Nodal	NM_013611	194	GAGGGCCCACTCACCATTGACATT	TCCAGTGCCCTGGGGTCCTTTAG
Eomes	NM_010136	204	GGCGCATGTTTCCTTTCTTGAGC	AGTGGGAGCCAGTGTTAGGAGATTCTG
Gata6	NM_010258	161	TGGCGTAGAAATGCTGAGGGTGAG	CTGTTACCGGAGCAAGCTTTTGACTTATT
Foxa2	NM_010446	164	CGCTCGGGACCCCAAGACATAC	TCTGCCGGTAGAAAGGGAAGAGGTC
Cer1	NM_009887	143	CTGCCCATCAAAAGCCACGAAGTA	CCGGGAAAACGAATGGAACTGC
Sox17	NM_011441	60	CGGCCGGCACCTACACTTACG	CGGGCGGCTCTACGGACACT
T	NM_009309	151	TGCTTTCCCGAGACCCAGTTCATAG	AGTCCCCCGGTTCCTCCATTACAT
Fgf5	NM_010203	94	GGACAGAGGCCACCGCACACTAA	AATGAGGGCAGGGGGCAGATAAAA
Nes	NM_016701	288	CACCCCTCAACCCTCACCACTCTATTT	CTGGGGTCTCATGGTTCTGGGTTTT
Chrna2	NM_144803	86	CCACCGGAACCTATAACAGCAAGAAGT	CAGCCGGCGGATAACGAAGTAGTAG
Zcchc12	NM_028325	103	TCGCCTGCATCAGCTCCTGGTAG	GGGAAGGCGCTCTGGCTCATTT
Eno2	NM_013509	236	GTTTTACCGCGATGGCAAATACGACT	CGATGCGCTTGGGGTTGGTC
Npy	NM_023456	257	AAGCGAATGGGGCTGTGTGGACT	CTTGTTCTGGGGGCGTTTTCTGTG
Gata4	NM_008092	215	TGCCGAGGGTGAGCCTGTATGTAAT	TGCTGCTAGTGGCATTGCTGGAGT
Nkx2.5	NM_008700	156	GACCCTCGGGCGGATAAAAAAGAG	GCGCCGCTCCAGCTCGTAGA
Mef2c	NM_001170537	102	ATCTCCGCGTTCTTATCCCACCTG	CCACCGGGGTAGCCAATGACTG
Mlc2v	NM_010861	186	AGGGCTCAGTCCTTCTCTTCTCCGT	TCCCGAGGGCAAAGGGTCACT
aMhc	NM_010856	252	CGCATCAAGGAGCTCACCTACCAGA	GGCACCAATGTCCCGGCTCTT
Afp	NM_007423	195	CAAAGCTGCGCTCTCTACCAGACCTTAG	GTCGGCCATTCCCTCACCACAG

Hnf4a	NM_008261	178	ACGGCTGCAAGGGGTTCTTCAG	ATCCGGTCCCGCTCATTTTGG
Alb	NM_009654	136	ATGTCTTCCTGGGCACGTTCTTGTATG	CATGCGGGAGGATTGGCTTCAG
Cyp1a1	NM_009992	193	CCTTCCGGCATTCATCCTTCGTC	AGAGTGCCGCTGGGGGTGAGA

Table S1, Primer sequences, Related to Materials and Methods

Gene abbreviations, NCBI reference sequences of mRNAs (used as template for primer design), amplicon size and primer sequences for the Real-Time PCR analysis.

Supplementary References

1. Chae, H.-D. & Broxmeyer, H.E. SIRT1 deficiency downregulates PTEN/JNK/FOXO1 pathway to block reactive oxygen species-induced apoptosis in mouse embryonic stem cells. *Stem Cells Dev.* **20**, 1277-1285 (2011).

2. Lee, D.E. *et al.* 6,7,4'-trihydroxyisoflavone inhibits HCT-116 human colon cancer cell proliferation by targeting CDK1 and CDK2. *Carcinogenesis* **32**, 629-635 (2011).

3. Borel, F., Lacroix, F.B. & Margolis, R.L. Prolonged arrest of mammalian cells at the G1/S boundary results in permanent S phase stasis. *J. Cell Sci.* **115**, 2829-2838 (2002).

4. Brueckner, B. *et al.* Epigenetic reactivation of tumor suppressor genes by a novel small-molecule inhibitor of human DNA methyltransferases. *Cancer Res.* **65**, 6305-6311 (2005).

5. Peck, B. *et al.* SIRT inhibitors induce cell death and p53 acetylation through targeting both SIRT1 and SIRT2. *Mol. Cancer Ther.* **9**, 844-855 (2010).

6. Rong, Y., Doctrow, S.R., Tocco, G. & Baudry, M. EUK-134, a synthetic superoxide dismutase and catalase mimetic, prevents oxidative stress and attenuates kainate-induced neuropathology. *Proc. Natl. Acad. Sci. USA* **96**, 9897-9902 (1999).

7. Caserta, T.M., Smith, A.N., Gultice, A.D., Reedy, M.A. & Brown, T.L. Q-VD-OPh, a broad spectrum caspase inhibitor with potent antiapoptotic properties. *Apoptosis* **8**, 345-352 (2003).

8. Sawada, M., Hayes, P. & Matsuyama, S. Cytoprotective membrane-permeable peptides designed from the Bax-binding domain of Ku70. *Nat. Cell Biol.* **5**, 352-357 (2003).

9. Koledova, Z., Kafkova, L.R., Krämer, A. & Divoky, V. DNA damage-induced degradation of Cdc25A does not lead to inhibition of Cdk2 activity in mouse embryonic stem cells. *Stem Cells* **28**, 450-461 (2010).

10. Strom, E. *et al.* Small-molecule inhibitor of p53 binding to mitochondria protects mice from gamma radiation. *Nat. Chem. Biol.* **2**, 474-479 (2006).

11. Fuks, B. *et al.* In vitro properties of 5-(benzylsulfonyl)-4-bromo-2-methyl-3(2H)-pyridazinone: a novel permeability transition pore inhibitor. *Eur. J. Pharmacol.* **519**, 24-30 (2005).

12. Peixoto, P.M., Ryu, S.-Y., Bombrun, A., Antonsson, B. & Kinnally, K.W. MAC inhibitors suppress mitochondrial apoptosis. *Biochem. J.* **423**, 381-387 (2009).

13. Hur, W. *et al.* A small-molecule inducer of the antioxidant response element. *Chem. Biol.* **17**, 537-547 (2010).

14. Hayes, J.D. & Dinkova-Kostova, A.T. The Nrf2 regulatory network provides an interface between redox and intermediary metabolism. *Trends Biochem. Sci.* **39**, 199-218 (2014).

15. Takayama, T. *et al.* Sexually dimorphic expression of the novel germ cell antigen TEX101 during mouse gonad development. *Biol. Reprod.* **72**, 1315-1323 (2005).

16. Gill, M.E., Hu, Y.-C., Lin, Y. & Page, D.C. Licensing of gametogenesis, dependent on RNA binding protein DAZL, as a gateway to sexual differentiation of fetal germ cells. *Proc. Natl. Acad. Sci. USA* **108**, 7443-7448 (2011).

17. Suzuki, A. & Saga, Y. Nanos2 suppresses meiosis and promotes male germ cell differentiation. *Genes Dev.* **22**, 430-435 (2008).

18. Saba, R., Kato, Y. & Saga, Y. NANOS2 promotes male germ cell development independent of meiosis suppression. *Dev. Biol.* **385**, 32-40 (2014).

19. Suzuki, A., Igarashi, K., Aisaki, K.-I., Kanno, J. & Saga, Y. NANOS2 interacts with the CCR4-NOT deadenylation complex and leads to suppression of specific RNAs. *Proc. Natl. Acad. Sci. USA* **107**, 3594-3599 (2010).

20. Vacanti, N.M. & Metallo, C.M. Exploring metabolic pathways that contribute to the stem cell phenotype. *Biochim. Biophys. Acta* **1830**, 2361-2369 (2013).

21. Villeneuve, N.F., Sun, Z., Chen, W. & Zhang, D.D. Nrf2 and p21 regulate the fine balance between life and death by controlling ROS levels. *Cell Cycle* **8**, 3255-3256 (2009).

22. Zhang, J., Nuebel, E., Daley, G.Q., Koehler, C.M. & Teitell, M.a. Metabolic Regulation in Pluripotent Stem Cells during Reprogramming and Self-Renewal. *Cell Stem Cell* **11**, 589-595 (2012).

23. Esakky, P., Hansen, D.A., Drury, A.M. & Moley, K.H. Molecular analysis of cell type-specific gene expression profile during mouse spermatogenesis by laser microdissection and qRT-PCR. *Reprod Sci.* **20**, 238-252 (2013).

24. Datta, K.M., Dasgupta, J., Sengupta, T. & De, S. Glycogen metabolism in human fetal testes. *J. Biosci.* **13**, 117-121 (1988).

25. Villarroel-Espíndola, F. *et al.* Muscle glycogen synthase isoform is responsible for testicular glycogen synthesis: glycogen overproduction induces apoptosis in male germ cells. *J. Cell. Biochem.* **114**, 1653-1664 (2013).
